# 3D Computational Modeling of Defective Early Endosome Distribution in Human iPSC-Based Cardiomyopathy Models

**DOI:** 10.3390/cells13110923

**Published:** 2024-05-27

**Authors:** Hafiza Nosheen Saleem, Nadezda Ignatyeva, Christiaan Stuut, Stefan Jakobs, Michael Habeck, Antje Ebert

**Affiliations:** 1 Heart Research Center Goettingen, Department of Cardiology and Pneumology, University Medical Center Goettingen, Georg-August University of Goettingen, 37077 Goettingen, Germany; 2 DZHK (German Center for Cardiovascular Research), Partner Site Goettingen, 37075 Goettingen, Germany; 3 Research Group Mitochondrial Structure and Dynamics, Department of NanoBiophotonics, Max Planck Institute for Multidisciplinary Sciences, 37077 Goettingen, Germany; 4 Clinic of Neurology, High Resolution Microscopy, University Medical Center Goettingen, 37075 Goettingen, Germany; 5 Fraunhofer Institute for Translational Medicine and Pharmacology ITMP, Translational Neuroinflammation and Automated Microscopy, 37075 Goettingen, Germany; 6 Microscopic Image Analysis, 39065 Jena University Hospital, Kollegiengasse 10, 07743 Jena, Germany

**Keywords:** endosomes, human iPSCs, computational modelling, signal processing, signal transduction, STED, dilated cardiomyopathy, heart failure

## Abstract

Intracellular cargo delivery via distinct transport routes relies on vesicle carriers. A key trafficking route distributes cargo taken up by clathrin-mediated endocytosis (CME) via early endosomes. The highly dynamic nature of the endosome network presents a challenge for its quantitative analysis, and theoretical modelling approaches can assist in elucidating the organization of the endosome trafficking system. Here, we introduce a new computational modelling approach for assessment of endosome distributions. We employed a model of induced pluripotent stem cell-derived cardiomyocytes (iPSC-CMs) with inherited mutations causing dilated cardiomyopathy (DCM). In this model, vesicle distribution is defective due to impaired CME-dependent signaling, resulting in plasma membrane-localized early endosomes. We recapitulated this in iPSC-CMs carrying two different mutations, TPM1-L185F and TnT-R141W (MUT), using 3D confocal imaging as well as super-resolution STED microscopy. We computed scaled distance distributions of EEA1-positive vesicles based on a spherical approximation of the cell. Employing this approach, 3D spherical modelling identified a bi-modal segregation of early endosome populations in MUT iPSC-CMs, compared to WT controls. Moreover, spherical modelling confirmed reversion of the bi-modal vesicle localization in RhoA II-treated MUT iPSC-CMs. This reflects restored, homogeneous distribution of early endosomes within MUT iPSC-CMs following rescue of CME-dependent signaling via RhoA II-dependent RhoA activation. Overall, our approach enables assessment of early endosome distribution in cell-based disease models. This new method may provide further insight into the dynamics of endosome networks in different physiological scenarios.

## 1. Introduction

Human induced pluripotent stem cell-derived cardiomyocytes (iPSC-CMs) offer reproducible access to cardiomyocytes for molecular functional studies in large quantities [1,2]. While iPSC-CMs represent an early developmental stage of cardiac cells, they recapitulate key features of this cell type, such as contractile force generation. Importantly, human iPSCs present a convenient platform for site-specific genome editing using CRISPR/Cas9 as their proliferation is not limited. In the past decade, human iPSC-CMs were successfully applied to understand basic functions of cells and their dysfunction in cardiac diseases such as LEOPARD syndrome [3], Timothy syndrome [4], long QT syndrome [5,6,7], familial hypertrophic cardiomyopathy [8], and familial dilated cardiomyopathy (DCM) [9]. More recently, iPSC-CM disease models revealed molecular dysfunctions in essential processes such as cytoskeleton function and clathrin-mediated endocytosis (CME)-dependent cargo uptake and distribution [10,11]. Human iPSC-CMs from patients carrying inherited DCM mutations in sarcomere proteins displayed defective interactions of sarcomeres with other cytoskeleton elements as well as the plasma membrane [10,11]. This patho-mechanism presents the basis for defective F-actin polymerization as well as phosphatidylinositol 4,5-bisphosphate (PIP2) localization at the plasma membrane of DCM iPSC-CMs. Consequently, CME-dependent uptake of critical cargo as well as its distribution via early endosomes is impaired in the presence of DCM mutations [11]. This molecular patho-phenotype is rescued by activation of the CME-dependent signaling pathway, such as by stimulation of the RhoA GTPase via the small peptide Rho activator II (RhoA II), in DCM iPSC-CMs as well as live cardiomyocytes from adult patients [11]. The resulting recovery of CME-dependent signaling corrects the defective localization of early endosomes in MUT iPSC-CMs [11]. Early endosome distribution in healthy cells is well known to occur in a continuous manner, to ensure regular distribution of cargo within the cell [12,13,14]. There are no mathematical models available to describe altered positioning of vesicles in iPSC-CMs due to the presence of DCM mutations.

Here, we applied classification of normalized distance distributions with a sphericity-based model of the cell to describe different endosome dispositions in DCM (MUT) and WT control iPSC-CMs. As a basis for computational modelling, we employed single-cell 3D confocal z-stacks of Early Endosome Antigen 1 (EEA1)-positive early endosomes. We confirmed our findings with MUT iPSC-CMs carrying two different DCM mutations in sarcomere proteins, tropomyosin (TPM1)-L185F and troponin T (TnT)-R141W [11,15]. Both DCM mutations were characterized previously regarding sarcomere dysfunction, pathological CME-dependent signaling, and the resulting impaired disposition of early endosomes [11]. Three-dimensional (3D) spherical modelling of distance distributions revealed a bi-modal grouping of early endosomes in MUT iPSC-CMs, compared to WT controls. The bimodal organization of endosome populations in the presence of DCM mutations accurately reflected the arrest of vesicles at the plasma membrane in MUT iPSC-CMs due to defective CME-dependent signaling [11]. We corroborated our 3D spherical modelling analysis performed based on confocal imaging data arrays by a second approach, diffraction-unlimited STED super-resolution microscopy data for EEA1-positive endosomes in MUT iPSC-CMs. Moreover, our computational modelling approach correctly recapitulated recovery of defective endosome distribution following treatment with RhoA II in MUT iPSC-CMs. Analysis of spherical distance vesicle distributions in TnT-R141W iPSC-CMs revealed a reversion of the bimodal positioning of vesicles following activation of CME-dependent signaling by RhoA II. Together, our mathematical modeling approach for altered spatial distribution of endosomes in DCM iPSC-CM models may benefit in the future different types of mechanistic studies, but also disease modelling and the potential development of diagnostic tools for cardiac diseases.

## 2. Materials and Methods

Further details on Methods are available in the Supplementary Materials.

### 2.1. Generation, Culture, CRISPR/Cas9 Gene Editing, and Cardiac Differentiation of Human iPSCs

All protocols for studies with human iPSCs were approved by the Goettingen University Ethical Board (15/2/20 and 20/9/16An) and the Odense University Ethical Board (Projekt ID S-20140073HLP). Informed consent was obtained from all participants and all research was performed in accordance with relevant guidelines and regulations. WT1 iPSCs were derived and characterized as described before [11]. Human WT2 iPSCs described in [9] were a kind gift from Joseph C. Wu (Stanford University, Stanford, CA, USA). WT1 and WT2 represent the isogenic WT controls for the CRISPR/Cas9 mutation-introduced iPSC lines MUT1 (DCM TPM1-L185F) and MUT2 (DCM TnT-R141W). The CRISPR/Cas9-based generation of MUT1 iPSCs carrying the DCM mutation TPM1-L185F and MUT2 iPSCs carrying the DCM mutation TnT-R141W has been described before [11,15]. Human induced pluripotent stem cells (iPSCs) were grown on Matrigel-coated plates (ES qualified, BD Biosciences Franklin Lakes, NJ, USA) using chemically defined E8 medium as described previously [2,16]. Small molecule-based differentiation of human iPSCs into iPSC-CMs was performed as reported previously [17,18]. From day 7 of cardiac differentiation, spontaneously contracting iPSC-CMs were observed. Human iPSC-CMs were cultured in RPMI medium (Life Technologies, Carlsbad, CA, USA) complemented with B27 supplement (Life Technologies). On cardiac differentiation day 20–25, iPSC-CMs were dissociated with TryplE (Life Technologies, Carlsbad, CA, USA) and used for the respective experimental analysis. When indicated, MUT iPSC-CMs were treated with 3 μg/mL Rho activator II (RhoA II) (Cytoskeleton Inc., Denver, CO, USA) overnight.

### 2.2. STED Imaging

STED super-resolution microscopy was performed using either a STED 775 QUAD scanning microscope (Abberior Instruments, Goettingen, Germany) equipped with a Katana 08 HP 775 nm STED laser (Onefive GmbH, Regensdorf, Switzerland) and a UPlanSApo 100x/1.40 Oil objective (Olympus, Tokyo, Japan) or a Facility Line scanning microscope (Abberior Instruments, Goettingen, Germany) equipped with a 775 nm STED laser (Abberior Instruments, Goettingen, Germany) and a UPlanXApo 60x/1.42 Oil objective (Olympus) objective. Abberior STAR RED (Abberior Instruments, Goettingen, Germany) was excited at 640 nm. Images were recorded with a pixel size of 20 nm.

### 2.3. Vesicular Distribution Computational Modeling

All methods for computational modeling were implemented in Python3 based on the publicly available packages NumPy, SciPy and Matplotlib. The background of the confocal images was fitted with a Gaussian model whose parameters (mean and standard deviation) are estimated as described in the main text. The computation of scaled distance distributions was done using NumPy arrays and functions.

### 2.4. Statistical Analysis

Statistical significance was estanlished using GraphPad Prism v.8.4.2 (GraphPad Software Inc., San Diego, CA, USA). Unless indicated otherwise, n = 2 independent experiments were performed using independent batches of iPSC-cardiac differentiation for each experiment. Data are presented as mean ± standard error of mean (SEM).

## 3. Results

### 3.1. Early Endosomes Fail to Distribute in iPSC-CMs Carrying DCM Mutations

**Figure 1:**
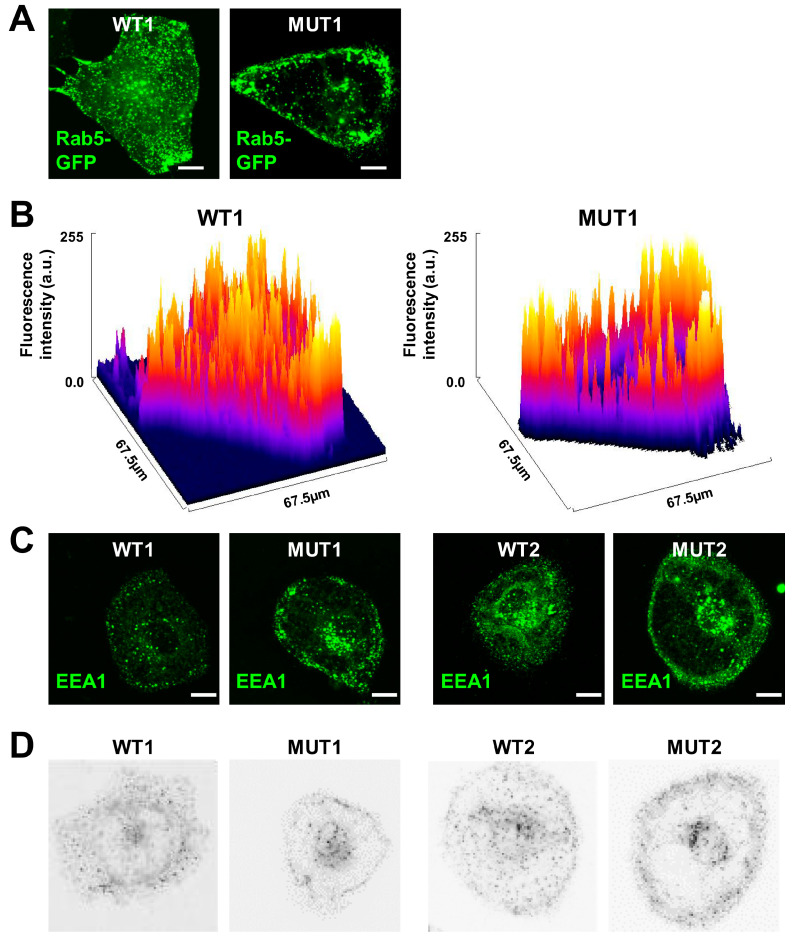
Defective distribution of early endosomes in DCM (MUT) iPSC-CMs. (**A**) Representative confocal images of Rab5-GFP overexpressing TPM1-L185F (DCM, MUT1) iPSC-CMs vs WT controls (WT1). Scale bar, 10 μm. (**B**) 3D surface plots of Rab5-GFP fluorescence intensities generated from images shown in (**A**). (**C**) 3D deconvoluted z-stacks of EEA1-positive early endosomes in MUT1 iPSC-CMs vs. WT controls. Scale bar, 20 μm. (**D**) Averages over image z-layers following threshold application show distribution of early endosomes in MUT1 iPSC-CMs and WT controls. Representative results are shown.

To investigate the molecular basis for pathological mis-localization of early endosomes in the presence of DCM mutations, we had previously generated CRISPR/Cas9 gene-edited iPSC lines carrying DCM mutations [11,15]. CRISPR/Cas9 genome-introduced iPSC lines carrying the DCM mutations TPM1-L185F (MUT1) [11] or TnT-R141W (MUT2) [15] were employed for small molecule-based differentiation into beating iPSC-cardiomyocytes (iPSC-CMs) according to standard protocols [16,17,19]. Human iPSC-CMs (WT1, MUT1, WT2, MUT2) displayed regular expression of the cardiac markers TnT, sarcomeric-α actinin, and myosin light chain 2a (Figure S1). Due to defective CME-dependent signaling in DCM iPSC-CMs, early endosomes do not distribute homogeneously following CME-dependent cargo uptake but rather remain localized adjacent to the plasma membrane in DCM (MUT) iPSC-CMs [11]. To recapitulate the molecular patho-phenotype of plasma membrane-arrested early endosomes in the presence of DCM mutations, we employed overexpression of Rab5-GFP in TPM1-L185F iPSC-CMs (MUT1) (Figure 1A). Indeed, MUT iPSC-CMs displayed an increase of plasma membrane-localized Rab5-positive early endosomes at the expense of the cytoplasmic pool of early endosomes, compared to WT controls (Figure 1A). To indicate the differential distribution of Rab5-GFP within MUT and WT iPSC-CMs in more detail, 3D surface plots were used (Figure 1B). To set up an experimental readout for computational modelling analysis, we employed detection of endogenous Early Endosome Antigen 1 (EEA1), an early endosome marker, in TPM1-L185F (MUT1) and TnT-R141W (MUT2) iPSC-CMs versus isogenic WT controls (WT1, WT2) (Figure 1C).

For quantitative analysis of vesicle distribution defects in MUT iPSC-CMs vs. WT controls, 3D confocal z-stacks were acquired. Averages over the image layers for EEA1-positive 3D confocal data in WT (WT1, WT2) and MUT (MUT1, MUT2) iPSC-CM groups were generated as representative results (Figure 1D) as well as for full data sets (Figure S2). Based on these data, differences in vesicular distributions were calculated for MUT1 versus WT1 iPSC-CMs (Videos S1 and S2).

### 3.2. Analysis of Normalized Distance Distributions for DCM/MUT vs. WT

**Figure 2:**
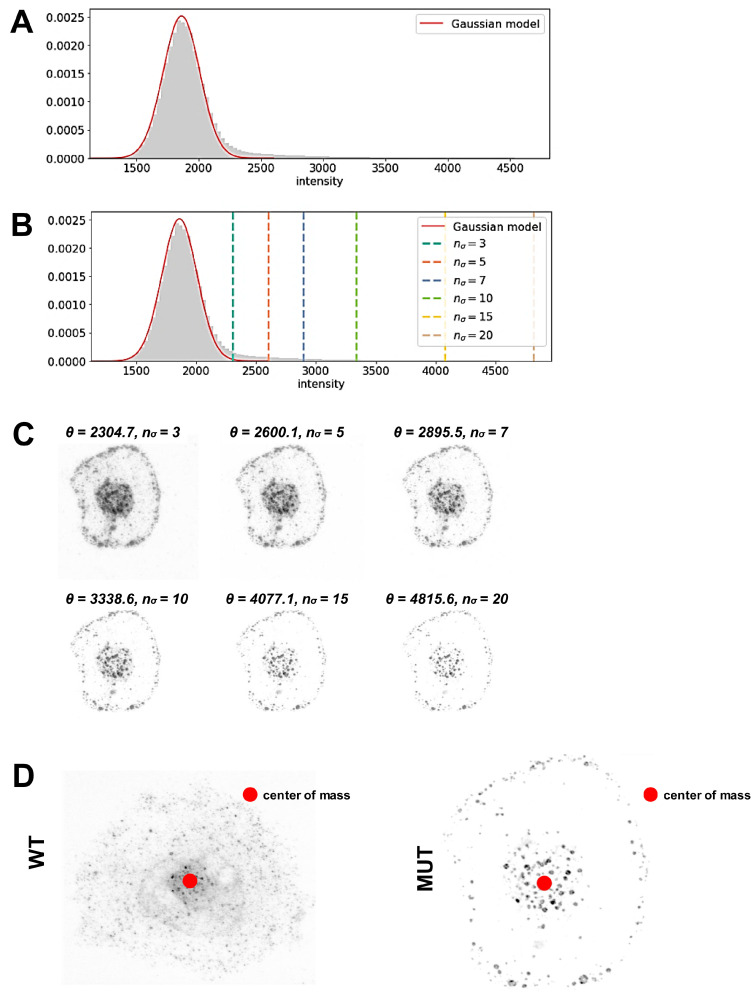
Analysis of normalized distance distributions of events in MUT vs WT iPSC-CMs. (**A**,**B**) Histogram distribution of pixel intensities. Data are shown as mean ± SD of background intensity: 1861.63 ± 147.70; estimated fraction of background pixels: 93.0%. n_ϭ_, adjustable parameters. (**C**) Binarized images shown for a representative cell (MUT iPSC-CM) with different thresholds applied as indicated for each image. (**D**) Estimation of the cell center based on the center of mass for thresholded images. A representative image is shown following application of thresholding as in (**A**–**C**).

First, to fit the image background, histograms of intensities over all z-layers were computed (Figure 2). A custom algorithm fits a Gaussian model to the distribution of background intensities, treating the signals as outliers residing in the right tail of the distribution. The algorithm identifies pixels that are likely to carry a true fluorescence signal based on a threshold derived from parameters of the Gaussian distribution. The center of the Gaussian peak is estimated by determining the location of the maximum of the intensity histogram. The variance of the Gaussian distribution was estimated by computing the mean squared deviation of intensities smaller than the center of the Gaussian peak (Figure 2A). Only intensities smaller than the center were considered to avoid contributions from the true signal included in the far right tail of the intensity histogram. We assumed that for the mean μ and the standard deviation σ of the Gaussian distribution modelling the background fluorescence, the given threshold *θ* was of the form: *θ = μ + n_σ_σ* where *n_σ_* is an adjustable parameter (Figure 2B). In this study, we typically employed *n_σ_* = 10 which is a very conservative choice. However, the features that we evaluated here were largely unaffected by the exact choice of *n_σ_*. This is indicated by binarized images (Figure 2C) obtained with different thresholds indicated for each binarized image (Figure 2C).

### 3.3. Spherical Modeling Recapitulates Altered Vesicular Distributions in MUT iPSC-CMs

We next sought to select a model that would enable the description of different distance distributions for vesicle (event) locations in WT or MUT cells, using binarized images (Figure 2C). As described before, early endosomes no longer distribute uniformly in MUT cells but instead arrest at the plasma membrane or accumulate in perinuclear regions (Figure 1A–C). Therefore, we expected a bimodal distribution for the distances of vesicles (events), corresponding to signals given in pixels when starting from the center of mass (Figure 2D).

To compute the distances of events, we employed an estimation of the cell center based on the center of mass for thresholded images as an estimate of the central position (Figure 2D):

$$c=\frac{\sum_{i}[y_{i}>|\theta ]\left(y_{i}-\theta \right)x_{i}}{\sum_{i}[y_{i}>\theta ]\left(y_{i}-\theta \right)}$$

The index *i* enumerates all pixels with intensities *y_i_* and pixel centers *x_i_*. The Iverson bracket is denoted as [ ], i.e., [*y_i_* > *θ*] is one if *y_i_* is indeed greater than the threshold, or otherwise zero (Figure 2C,D). 

### 3.4. Analysis of Distance Distributions for Vesicular Populations Using Spherical Modelling

**Figure 3:**
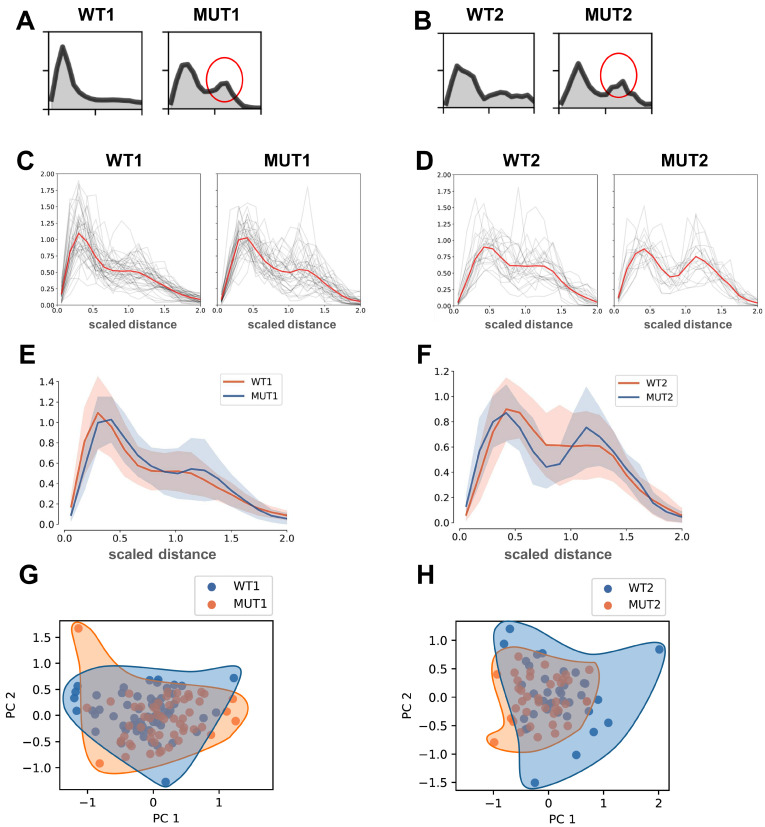
Spherical modelling enables analysis of distance distributions of early endosome populations. (**A**,**B**) Weighted histograms after computing distances for all pixels from the center of mass. Representative histograms are shown. The increased plasma membrane-localized population of early endosome vesicles is present in MUT iPSC-CMs (red circle) but not in WT controls where early endosomes distribute more homogenously within the cytoplasm. (**A**) TPM1-L185F (MUT1) iPSC-CMs versusand WT controls (WT1); (**B**) TnT-R141W (MUT2) iPSC-CMs versus WT controls (WT2). (**C**,**D**) Spherical scaled distance distributions for the entire data population indicates a bi-modal distribution for MUT iPSC-CMs compared to WT controls. Shown in red is the average of individual spherical scaled distance distributions (in grey). (**C**) TPM1-L185F (MUT1) iPSC-CMs versus WT controls (WT1); WT1, n = 60 cells, MUT1, n = 56 cells. (**D**) TnT-R141W (MUT2) iPSC-CMs versus WT controls (WT2); WT2, n = 38 cells, MUT2, n = 38 cells. (**E**,**F**) Averaged scaled distance distributions for the entire data population shown in (**C**) for MUT iPSC-CMs versus WT controls. (**E**) TPM1-L185F (MUT1) iPSC-CMs and WT controls (WT1); (**F**) TnT-R141W (MUT2) iPSC-CMs versus WT controls (WT2). (**G**,**H**) Principal component analysis (PCA) for data (shown in (**A**–**F**)) from MUT iPSC-CMs versus WT controls. (**G**) TPM1-L185F (MUT1) iPSC-CMs versus vs. WT controls (WT1); (**H**) TnT-R141W (MUT2) iPSC-CMs versus WT controls (WT2).

We applied this model to image data for WT and MUT groups. Distances of all pixels from the cell center were computed and formed into distance histograms (Figure 3A,B) where the distance of each vesicular event was weighted by its corresponding intensity:

$$\omega_{i}\propto \left[y_{i}>\theta \right]\left(y_{i}-\theta \right)$$

The weight of pixels below the threshold *θ* is zero, therefore these pixels did not contribute to the distance distributions. Distances for all pixels from the center of mass were calculated and included in distance distribution histograms for WT and MUT cells (Figure 3A). Indeed, we found in MUT iPSC-CMs a bimodal distribution of vesicles, in which the first peak stems from vesicles located in perinuclear areas, while the second peak corresponds to plasma membrane-localized vesicles (Figure 3A), as expected based on confocal images (Figure 1A–C). In contrast, the vesicular distribution in WT iPSC-CMs was more homogenous and characterized by the absence of the second peak corresponding to an enrichment of plasma membrane-localized early endosomes (Figure 3A), as anticipated based on confocal images (Figure 1A–C). We confirmed the bimodal distribution also for MUT iPSC-CMs carrying the second mutation, TnT-R141 (MUT2), compared to isogenic WT controls (WT2) (Figure 3B).

### 3.5. Robustness of Distance Distributions Using 3D Spherical Modeling

To further test the spherical modeling approach, we next sought to establish its robustness against different choices of thresholds (Figure S3A). We found that the overall shape of the distance distribution is largely unaffected by the choice of the threshold (Figure S3A). Moreover, we tested the robustness of the method against sampling density (Figure S3B). To speed up calculations, we down-sampled images in each layer from their original size of 1024 × 1024 to 512 × 512 pixels or fewer. Down-sampling was found to not affect the shape of the distance distribution (Figure S3B). Therefore, we subsequently used a sampling rate of 2 (i.e., stacks of images with 512 × 512 pixels) and employed n_σ_ = 10 to determine the threshold.

### 3.6. Scaled Distancing of 3D Spherical Models Accurately Reflects Altered Distribution of Early Endosomes in MUT iPSC-CMs

We considered variations in cell size as a factor contributing to the accuracy of spherical 3D modeling of vesicular distributions in both WT and MUT groups. Therefore, we sought to compare distance distributions across differently sized cells (Figure S4A). As expected, the scaling of the distance distributions (Figure S4B) was found to be dependent on the size of the cell (Figure S4A,B). To account for these differences in size, we estimated the size of the cell by computing the radius of gyration:

$$R_{g}=\sqrt{{\sum_{i}\omega }_{i}\|x_{i}-c{\|}^{2}/\sum_{i}\omega_{i}}$$

As before, it was $\omega_{i}=\left[y_{i}>\theta \right]\left(y_{i}-\theta \right)\;$and *c* represented the center of mass of the selected pixels. Applying this, we then computed the weighted distribution histograms over the scaled distances: $\|x_{i}-c\|/R_{g}$ (Figure S4C). Using the scaled distance distribution for 3D spherical modeling, we computed all distributions of the scaled distances for WT (WT1, WT2) and MUT (MUT1, MUT2) groups (Figure S5A–D). The majority of MUT iPSC-CMs carrying two different DCM mutations (MUT1, MUT2) presented with a bimodal distribution of vesicles (Figure S5B,D) while this was the exception for WT iPSC-CMs (WT1, WT2) which showed a more homogenous distribution of early endosomes (Figure S5A,C). To compare the overview of scaled distance distributions over the entire data population, we plotted all vesicular distributions of WT1 vs MUT1 (Figure 3C) as well as WT2 vs. MUT2 (Figure 3D). Due to the large standard deviation in human iPSC-CM models [20], the differential distribution of vesicular populations for all 3D confocal images of MUT iPSC-CMs compared to WT is not statistically significant. However, even when plotting all data for WT and MUT groups, the bi-modal distribution indicating an increase of the plasma membrane-adjacent population of early endosomes in the MUT group becomes apparent (Figure 3C,D), particularly for MUT2 vs. WT2 (Figure 3D) and when comparing overlays of WT1 and MUT1 (Figure 3E) as well as WT2 and MUT2 (Figure 3F). To test if the observed differences were due to cell size-based adaptation of scaled distance distributions, we also analyzed scaled distances for all events without considering cell size (Figure S6). Even if we did not account for differences in the cell size within WT and MUT groups, an enrichment of large distances in the endosome populations was observed for MUT iPSC-CMs (MUT1, MUT2), but not for WT (WT1, WT2) (Figure S6A–D).

To get a better overview of the trends in the distance distributions for MUT iPSC-CMs compared to WT controls, we used principal component analysis (PCA). We observed a modest separation along the first principal component (Figure 3G,H). The PCA of scaled distance distributions confirmed different segregation of vesicle populations in the WT1 vs. MUT1 (Figure 3G) and WT2 vs. MUT2 (Figure 3H) comparisons. Together, these findings indicate that spherical 3D modeling of vesicular population distributions is robust and confirms differential localization of early endosomes in MUT iPSC-CMs compared to WT controls.

### 3.7. STED Super-Resolution Microscopy Combined with 3D Spherical Modeling Enables More Accurate Quantification of Vesicular Content and Distribution

**Figure 4:**
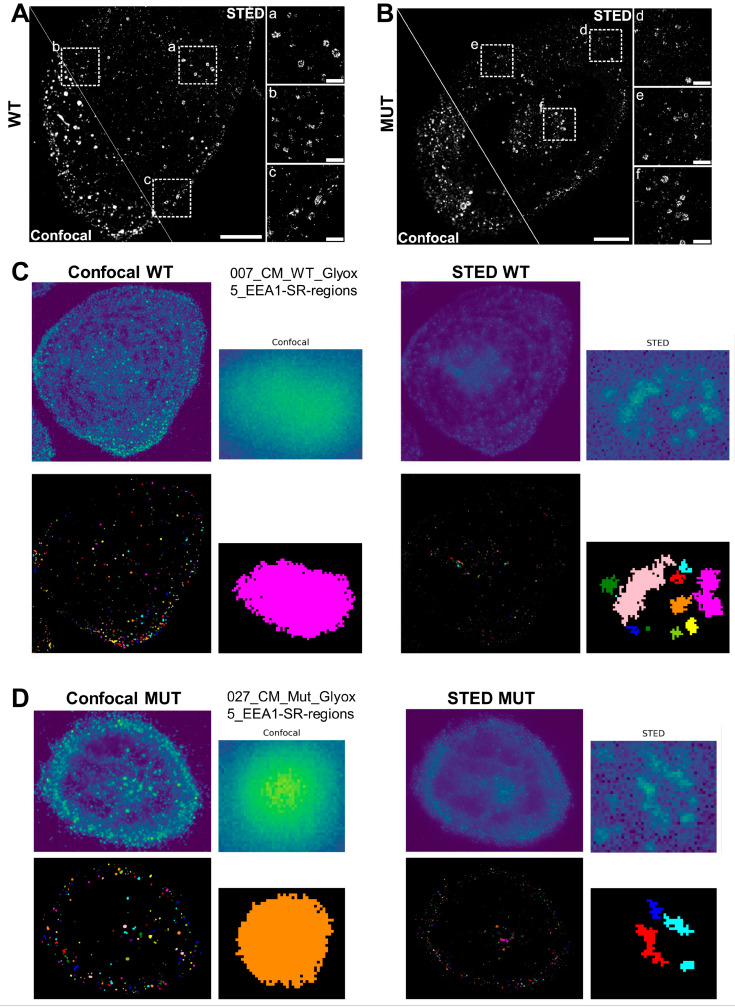
STED super-resolution microscopy for more accurate quantifications of vesicular content and distribution. (**A**,**B**) Comparison of confocal and STED microscopy showing the same ROI imaged first by confocal, subsequently by STED microscopy, for WT2 (**A**) and MUT2 (**B**) iPSC-CMs; representative images are shown. The border between the confocal (left) and STED (right) image is shown as a line, respectively. Scale bar, 10 µm. (**a**–**c**): Magnifications of areas indicated in overview WT STED image. Scale bar, 2 µm. (**d**–**f**): Magnifications of areas indicated in overview MUT STED image. Scale bar, 2 µm. (**C**,**D**) Determining the center of mass and the gyration radius for STED imaged vs. confocal imaged ROIs, for WT2 (**C**) and MUT2 (**D**) iPSC-CMs prior to computational modeling for detection and quantification of vesicles (events). Areas detected in confocal imaging as one large ROI are resolved into individual events by STED microscopy.

Despite the application of 3D spherical modeling to accurately describe the distributions of vesicular populations, we reasoned our previous data may be restricted by the limited resolution of confocal-based 3D imaging. Therefore, we applied STED super-resolution microscopy in comparison with confocal imaging (Figure 4A,B). STED-based imaging enabled us to capture substantially more detail than confocal imaging for resolving individual vesicles (Figure 4B, inlets) in both WT and MUT iPSC-CMs. This was confirmed when applying the spherical modelling for comparative detection of early endosomes (events) (Figure 4C,D). Thresholding of STED images was established using:

threshold = mean-intensity + n_sigma × stdev-intensity

**Figure 5:**
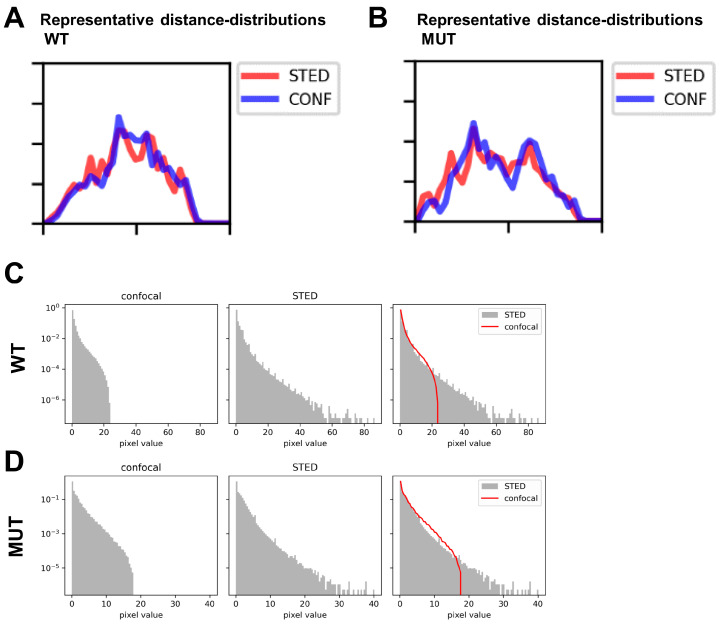
Scaled distances and intensity distributions for STED data populations of MUT iPSC-CMs vs. WT. (**A**,**B**) Representative scaled distance distributions for the same ROI imaged via STED (red) vs. confocal (blue) microscopy, applying the 1sigma criterion, comparing WT (**A**) and MUT (**B**) iPSC-CMs. (**C**,**D**) Intensity distributions for all events detected via STED imaging (grey histograms) compared to the range of events imaged by confocal microscopy (red line in overlay) for the same ROI. Results from a representative cell are shown for WT (**C**) and MUT iPSC-CMs (**D**).

While confocal optics frequently detected larger areas of signal, STED imaging of the same cell’s ROI allowed us to resolve these areas into individual events (Figure 4C,D, inlets). On the other hand, the relative distance distributions detected by STED and confocal imaging in WT vs. MUT iPSC-CMs was comparable (Figure 5A,B and Figure S7A,B). However, when assessing relative intensity distributions of events in WT vs. MUT iPSC-CMs, the range of STED-detected events was substantially larger than for the same ROI imaged via confocal optics (Figure 5C,D and Figure S8A,B).

### 3.8. 3D Spherical Modeling Confirms Recovery of Vesicular Distributions in MUT iPSC-CMs following RhoA II Treatment

**Figure 6:**
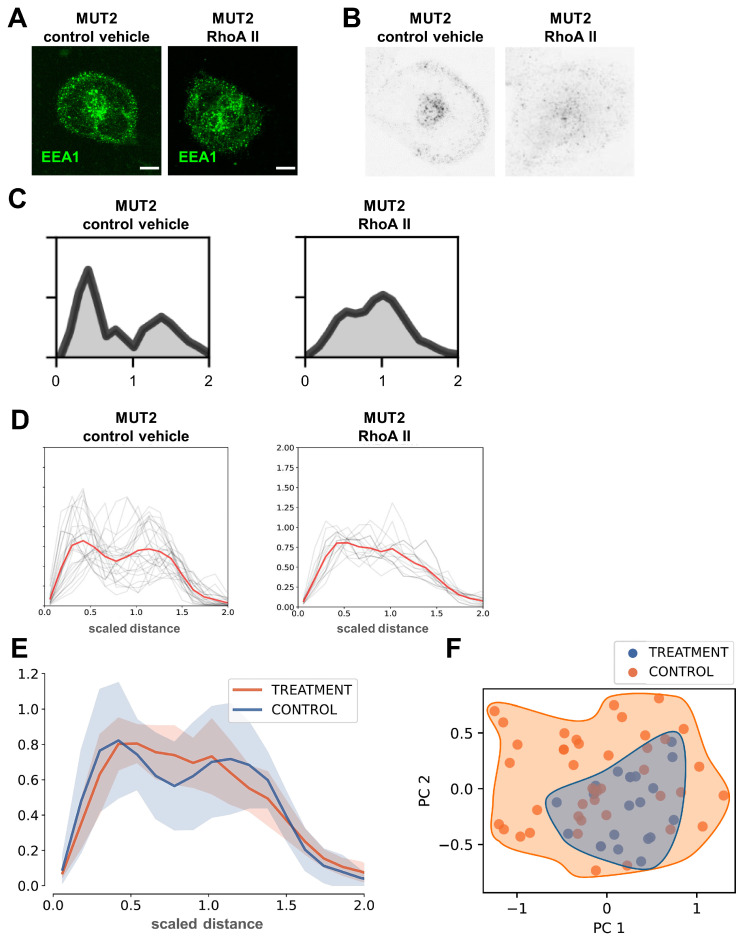
3D spherical modelling confirms recovery of vesicular distributions in MUT iPSC-CMs following RhoA II treatment. (**A**) Distribution of EEA1-positive early endosomes in MUT2 iPSC-CMs following RhoA II treatment versus control vehicle. Representative images are shown; scale bar, 20 μm. (**B**) Averages over the image z-layers following threshold application as in Figure 1D. Representative results are shown for RhoA II-treated MUT2 iPSC-CMs compared to control vehicle. (**C**) Weighted distance distribution histograms for MUT2 iPSC-CMs treated with RhoA II or control vehicle. Representative results are shown. (**D**) Spherical scaled distance distributions for full data populations indicate a reduced bi-modal distribution in RhoA II-treated MUT2 iPSC-CMs compared to control vehicle. Control vehicle, n = 42 cells; RhoA II, n = 41 cells. Orange and blue lines represent the overlay of scaled distance distributions for the RhoA II treatment and control vehicle groups, respectively. (**E**) Averages of scaled distance distributions for RhoA II-treated MUT2 iPSC-CMs versus control vehicle. (**F**) PCA analysis of RhoA II-treated data (shown in (**D**,**E**)) from MUT2 iPSC-CMs versus control vehicle.

Previously, it was demonstrated that defective CME-dependent cargo uptake and distribution in the presence of DCM mutations is recovered in the presence of a small-molecule peptide activating the RhoA GTPase, RhoA activator II (RhoA II) [11]. We utilized this to further challenge the 3D spherical modelling method, and we tested whether reversion of the vesicular distribution defect in MUT iPSC-CMs using RhoA II treatment would be recapitulated by our computational modeling approach. MUT iPSC-CMs carrying the DCM mutation TnT-R141W (MUT2) were treated with RhoA II or control vehicle, followed by 3D confocal analysis of EEA1-positive early endosomes (Figure 6A). Subsequently, spherical modeling of scaled distance distributions was applied to RhoA II and control vehicle groups. Thresholds for 3D confocal images were set according to the same criteria as before (Figure 1C,D) for RhoA II or control vehicle-treated MUT2 iPSC-CM image data (Figure 6B and Figure S9A,B).

Next, distances for all pixels were extracted depending on their signal intensities, starting from the center of mass, and weighted histograms for spherical distance distributions were computed (Figure 6C and Figure S9C,D). We calculated the cell size based on gyration across different cells for RhoA II vs. control vehicle-treated MUT2 iPSC-CMs. Averages for scaled distance distributions for all cell populations from RhoA II vs. control vehicle groups were plotted (Figure 6D and Figure S9A–D). Indeed, these results showed a substantial recovery of homogenous intracellular distribution of early endosomes in RhoA II-treated MUT iPSC-CMs, compared to control vehicle (Figure 6D). Likewise, when overlaying scaled distance distribution averages from RhoA II and control vehicle-treated groups, RhoA II-treated cells recapitulated a substantial loss of the bimodal distribution, represented by a reduced plasma membrane-localized vesicle population in the RhoA II group (Figure 6A,E).

As a control, we plotted all un-scaled distance distributions for RhoA II vs. control vehicle-treated MUT2 iPSC-CMs (Figure S10A). When not considering cell size, a substantial recovery of the pathological bi-modal vesicular distribution was also observed in the RhoA II-treated MUT2 iPSC-CMs (Figure S10A). The same was noted for averages of the respective scaled distance distributions for all data from the RhoA II vs. control vehicle-treated MUT2 iPSC-CMs (Figure S10B). PCA analysis of spherically modelled scaled distance distributions also indicated some recovery of vesicle distribution after treatment with RhoA II (Figure 6F). Together, these findings suggest that 3D spherical modelling of scaled distance distributions describes defective intracellular arrangement of early endosomes in iPSC-CMs carrying DCM mutations vs. WT controls. Moreover, recovery of vesicle disposition following RhoA II-based treatment to rescue CME-dependent cargo distribution defects is accurately recapitulated by this computational modelling approach.

## 4. Discussion

Spatially defined compartments for cargo delivery within the cell are organized into different domains, and EEA1-positive endosomes present an early vehicle for distribution of cargo [12]. Previous modelling approaches for cargo delivery routing within the cell included the Rab family GTPase cascades, in particular the identity switch of the Rab5 to Rab7 endosome conversion along the degradative pathway [21]. Other studies employed theoretical framework inference to enable quantitative analysis of the endosomal system [22]. Due to its highly dynamic organization, the discrete nature of endosomal network patterns presents a challenge for theoretical modelling approaches. Here, we focused on steady-state distributions of early endosomes in iPSC-CMs carrying DCM mutations (MUT). In contrast to WT controls, which display homogenous dissemination of endosome cargo vehicles, MUT iPSC-CMs fail to distribute early endosomes regularly. Instead, MUT iPSC-CMs present with a plasma membrane-localized endosome population, at the expense of the cytoplasmic pool of early endosomes [11]. To describe this patho-physiological function, we used binarized and thresholded 3D image layers for computing distances of EEA1-positive vesicles based on a spherical estimation of the cell. Employing this approach, 3D spherical modelling of scaled distance distributions showed a bi-modal presentation in weighted histograms, recapitulating how endosome populations segregate into spatially distinct locations in MUT iPSC-CMs vs. WT controls. 3D spherical modelling of vesicle locations was unaffected by scaling distances based on cell size. We also applied spherical modelling of distance distributions to high-resolution STED microscopy images, revealing additional detail from the increasingly resolved depth of data compared to confocal imaging. Moreover, our findings confirmed the recovery of disease phenotypes such as impaired distribution of cargo carriers in DCM iPSC-CMs using the small RhoA GTPase-stabilizing peptide, RhoA II [11]. By applying spherical modelling of scaled distance distributions, we confirmed the bi-modal presentation of vesicle populations to be reverted, resulting in a restored homogenous early endosome dissemination within MUT iPSC-CMs.

It should be noted that scaled distancing of events in conjunction with spherical modelling of cells is limited by the rich variety of different 3D cell shapes which iPSC-CMs assume. Moreover, to apply our modelling approach to adult cardiomyocytes, this model would need to be adapted for alternate shapes of mature cardiac cells presenting with an elongated, rod-like shape [23]. For example, an ellipsoid coordinate system integrating segmentation would be expected to recapitulate an even more precise model. Nevertheless, using spherical modelling of scaled distance distributions, we recapitulated key features of spatially distinct endosome populations in MUT iPSC-CMs, compared to WT controls. Moreover, our computational 3D modelling platform, despite the simplified approach using a spherical approximation of the cells, is also highly sensitive, especially when applied to high-resolution STED imaging data.

Therefore, within the limitations of the system, we consider our approach a new tool and starting point for 3D modelling of vesicular compartments.

Seminal progress has been made in functional understanding of molecular dysfunctions in cardiac diseases using iPSC-based models [3,7,8,9]. These studies revealed numerous cardiac disease-specific molecular features which remain to be studied further to explore their full capacity for the development of new therapeutic directions. As research progresses and technologies such as super-resolution imaging develop rapidly [24,25,26], more accurate computer-based tools for modelling subcellular pathologies become instrumental to managing the huge amount of emerging information. The use of computational 3D spherical modelling not only delivers a defined description of molecular patho-phenotypes such as vesicular distribution defects in the presence of DCM mutations, but also reveals more detailed insights into disease mechanisms. Automated and accurate scientific analysis methods such as the one provided here also create a foundation for the implementation of integrative data platforms making use of machine learning and deep learning tools. We propose this new automated 3D computational modelling analysis to enable a more precise classification as well as quantification of DCM patho-phenotypes in pre-clinical human models. Future integration of different DCM mutations and additional imaging data types will enable the development of the 3D computational analysis platform further towards its translational application. Further on, multi-functional analysis algorithms may be integrated into the analysis platform for developing novel diagnostic tools that can employ large amounts of different types of data for predicting phenotypic outcomes of disease models. Ultimately, such diagnostic platforms may serve to assist the risk stratification in patient subpopulations, as well as support personalized treatment regimens in cardiac disease.

## 5. Conclusions

Our approach presents a new tool and starting point for automated 3D computational modelling of patho-phenotypes in disease models. We suggest this platform will assist to deliver a more precise classification as well as quantification of DCM disease phenotypes in pre-clinical human models. Onward, developing this platform further to integrate different imaging data types and multi-functional analysis algorithms will enhance its suitability for a translational application as a novel diagnostic tool. Initially predicting phenotypic outcomes of disease models, such diagnostic platforms may facilitate risk stratification in subgroups of patients with DCM.

## Data Availability

Data are contained within the article.

## Supplementary Materials

The following supporting information can be downloaded at: https://www.mdpi.com/article/10.3390/cells13110923/s1. Figure S1: Characterization of iPSC-CMs. Figure S2: Averages over z-layers (n = 25–60) for image-based data after threshold application. Figure S3: Robustness of distance distri-butions against sampling density. Figure S4: Dependence of distance distributions on cell size. Figure S5: Distance distributions for all WT and MUT data sets. Figure S6: All scaled distance distributions for WT and MUT data sets without spherical modelling. Figure S7: Distance distributions for STED imaging data from WT and MUT iPSC-CMs. Figure S8: Intensity distributions for all STED data populations of MUT iPSC-CMs vs. WT. Figure S9: Application of threshold and scaled distance distributions for MUT iPSC-CMs following RhoA II treatment. Figure S10: Scaled distance distributions for MUT iPSC-CMs following RhoA II treatment. Video S1: 3D movie of WT vesicular dis-tributions calculated based on confocal imaging of early endosome immunostaining in WT1 iPSC-CMs. Video S2: 3D movie of MUT vesicular distributions calculated based on confocal imaging of early endosome immunostaining in MUT1 iPSC-CMs.

## References

[1] Yamanaka S., Takahashi K. (2006). [Induction of pluripotent stem cells from mouse fibroblast cultures]. Tanpakushitsu Kakusan Koso.

[2] Chen G., Gulbranson D.R., Hou Z., Bolin J.M., Ruotti V., Probasco M.D., Smuga-Otto K., Howden S.E., Diol N.R., Propson N.E. (2011). Chemically defined conditions for human iPSC derivation and culture. Nat. Methods.

[3] Carvajal-Vergara X., Sevilla A., D’Souza S.L., Ang Y.S., Schaniel C., Lee D.F., Yang L., Kaplan A.D., Adler E.D., Rozov R. (2010). Patient-specific induced pluripotent stem-cell-derived models of LEOPARD syndrome. Nature.

[4] Yazawa M., Hsueh B., Jia X., Pasca A.M., Bernstein J.A., Hallmayer J., Dolmetsch R.E. (2011). Using induced pluripotent stem cells to investigate cardiac phenotypes in Timothy syndrome. Nature.

[5] Moretti A., Bellin M., Welling A., Jung C.B., Lam J.T., Bott-Flugel L., Dorn T., Goedel A., Hohnke C., Hofmann F. (2010). Patient-specific induced pluripotent stem-cell models for long-QT syndrome. N. Engl. J. Med..

[6] Itzhaki I., Maizels L., Huber I., Zwi-Dantsis L., Caspi O., Winterstern A., Feldman O., Gepstein A., Arbel G., Hammerman H. (2011). Modelling the long QT syndrome with induced pluripotent stem cells. Nature.

[7] Wang Y., Liang P., Lan F., Wu H., Lisowski L., Gu M., Hu S., Kay M.A., Urnov F.D., Shinnawi R. (2014). Genome editing of isogenic human induced pluripotent stem cells recapitulates long QT phenotype for drug testing. J. Am. Coll. Cardiol..

[8] Lan F., Lee A.S., Liang P., Sanchez-Freire V., Nguyen P.K., Wang L., Han L., Yen M., Wang Y., Sun N. (2013). Abnormal calcium handling properties underlie familial hypertrophic cardiomyopathy pathology in patient-specific induced pluripotent stem cells. Cell Stem Cell.

[9] Sun N., Yazawa M., Liu J., Han L., Sanchez-Freire V., Abilez O.J., Navarrete E.G., Hu S., Wang L., Lee A. (2012). Patient-specific induced pluripotent stem cells as a model for familial dilated cardiomyopathy. Sci. Transl. Med..

[10] Dai Y., Amenov A., Ignatyeva N., Koschinski A., Xu H., Soong P.L., Tiburcy M., Linke W.A., Zaccolo M., Hasenfuss G. (2020). Troponin destabilization impairs sarcomere-cytoskeleton interactions in iPSC-derived cardiomyocytes from dilated cardiomyopathy patients. Sci. Rep..

[11] Dai Y., Ignatyeva N., Xu H., Wali R., Toischer K., Brandenburg S., Lenz C., Pronto J., Fakuade F.E., Sossalla S. (2023). An Alternative Mechanism of Subcellular Iron Uptake Deficiency in Cardiomyocytes. Circ. Res..

[12] Zerial M., McBride H. (2001). Rab proteins as membrane organizers. Nat. Rev. Mol. Cell Biol..

[13] Wandinger-Ness A., Zerial M. (2014). Rab proteins and the compartmentalization of the endosomal system. Cold Spring Harb. Perspect. Biol..

[14] Hsu F., Spannl S., Ferguson C., Hyman A.A., Parton R.G., Zerial M. (2018). Rab5 and Alsin regulate stress-activated cytoprotective signaling on mitochondria. Elife.

[15] Xu H., Wali R., Cheruiyot C., Bodenschatz J., Hasenfuss G., Janshoff A., Habeck M., Ebert A. (2021). Non-negative blind deconvolution for signal processing in a CRISPR-edited iPSC-cardiomyocyte model of dilated cardiomyopathy. FEBS Lett..

[16] Ebert A.D., Kodo K., Liang P., Wu H., Huber B.C., Riegler J., Churko J., Lee J., de Almeida P., Lan F. (2014). Characterization of the molecular mechanisms underlying increased ischemic damage in the aldehyde dehydrogenase 2 genetic polymorphism using a human induced pluripotent stem cell model system. Sci. Transl. Med..

[17] Lian X., Hsiao C., Wilson G., Zhu K., Hazeltine L.B., Azarin S.M., Raval K.K., Zhang J., Kamp T.J., Palecek S.P. (2012). Robust cardiomyocyte differentiation from human pluripotent stem cells via temporal modulation of canonical Wnt signaling. Proc. Natl. Acad. Sci. USA.

[18] Lian X., Zhang J., Azarin S.M., Zhu K., Hazeltine L.B., Bao X., Hsiao C., Kamp T.J., Palecek S.P. (2013). Directed cardiomyocyte differentiation from human pluripotent stem cells by modulating Wnt/beta-catenin signaling under fully defined conditions. Nat. Protoc..

[19] Ebert A., Joshi A.U., Andorf S., Dai Y., Sampathkumar S., Chen H., Li Y., Garg P., Toischer K., Hasenfuss G. (2019). Proteasome-Dependent Regulation of Distinct Metabolic States During Long-Term Culture of Human iPSC-Derived Cardiomyocytes. Circ. Res..

[20] Matsa E., Burridge P.W., Yu K.H., Ahrens J.H., Termglinchan V., Wu H., Liu C., Shukla P., Sayed N., Churko J.M. (2016). Transcriptome Profiling of Patient-Specific Human iPSC-Cardiomyocytes Predicts Individual Drug Safety and Efficacy Responses In Vitro. Cell Stem Cell.

[21] Del Conte-Zerial P., Brusch L., Rink J.C., Collinet C., Kalaidzidis Y., Zerial M., Deutsch A. (2008). Membrane identity and GTPase cascades regulated by toggle and cut-out switches. Mol. Syst. Biol..

[22] Foret L., Dawson J.E., Villaseñor R., Collinet C., Deutsch A., Brusch L., Zerial M., Kalaidzidis Y., Jülicher F. (2012). A general theoretical framework to infer endosomal network dynamics from quantitative image analysis. Curr. Biol..

[23] Eschenhagen T., Mummery C., Knollmann B.C. (2015). Modelling sarcomeric cardiomyopathies in the dish: From human heart samples to iPSC cardiomyocytes. Cardiovasc. Res..

[24] Klar T.A., Jakobs S., Dyba M., Egner A., Hell S.W. (2000). Fluorescence microscopy with diffraction resolution barrier broken by stimulated emission. Proc. Natl. Acad. Sci. USA.

[25] Sahl S.J., Hell S.W., Jakobs S. (2017). Fluorescence nanoscopy in cell biology. Nat. Rev. Mol. Cell Biol..

[26] Stephan T., Bruser C., Deckers M., Steyer A.M., Balzarotti F., Barbot M., Behr T.S., Heim G., Hubner W., Ilgen P. (2020). MICOS assembly controls mitochondrial inner membrane remodeling and crista junction redistribution to mediate cristae formation. EMBO J..

