# Supplementary material for: 3D Computational Modeling of Defective Early Endosome Distribution in Human iPSC-Based Cardiomyopathy Models
Source: Cells. 2024 May 27;13(11):923. doi: 10.3390/cells13110923 (PMC11171759; doi:10.3390/cells13110923)

SUPPLEMENTARY MATERIALS

3D Computational Modeling of Defective Early Endosome Distribution in Human iPSC-based Cardiomyopathy Models

Hafiza Nosheen Saleem[1,2#], Nadezda Ignatyeva[1,2#], Christiaan Stuut[4],

Stefan Jakobs[4,5,6,7], Michael Habeck[3*], Antje Ebert[1,2*]

[1]Heart Research Center Goettingen, Department of Cardiology and Pneumology, University Medical Center Goettingen, Georg-August University of Goettingen, Goettingen, Germany; [2]DZHK (German Center for Cardiovascular Research), partner site Goettingen, Germany; [3]University Medical Center Jena, Germany; [4]Department of NanoBiophotonics, Research Group Mitochondrial Structure and Dynamics, Max Planck Institute for Multidisciplinary Sciences, Goettingen, Germany; [5]Clinic of Neurology, High Resolution Microscopy, University Medical Center Goettingen, Germany; [6]Fraunhofer Institute for Translational Medicine and Pharmacology ITMP, Translational Neuroinflammation and Automated Microscopy, Goettingen, Germany.

METHODS

Generation, culture, and CRISPR/Cas9 gene editing of human iPSCs. All protocols for studies with human iPSC were approved by the Goettingen University Ethical Board (15/2/20 and 20/9/16An) and the Odense University Ethical Board (Projekt ID S-20140073HLP). Informed consent was obtained from all participants and all research was performed in accordance with relevant guidelines and regulations. Human iPSCs from two different individuals were employed in this study. WT1 iPSCs were derived and characterized as described before (*1*). Human WT2 iPSCs described in (*2*) were a kind gift from Joseph C. Wu (Stanford University, CA). The iPSC lines have been deposited at the Goettingen University Medical Center Biobank (WT1) or the Stanford Cardiovascular Institute Biobank (WT2). WT1 and WT2 represent the isogenic WT controls for the CRISPR/Cas9 mutation-introduced iPSC lines MUT1 (DCM TPM1-L185F) and MUT2 (DCM TnT-R141W). The CRISPR/Cas9-based generation of MUT1 iPSCs carrying the DCM mutation TPM1-L185F and MUT2 iPSCs carrying the DCM mutation TnT-R141W was described before (*1, 3*). Human induced pluripotent stem cells (iPSCs) were grown on Matrigel-coated plates (ES qualified, BD Biosciences) using chemically defined E8 medium as described previously (*4, 5*). The culture medium was changed every day, and iPSCs were passaged every four days using EDTA (Life Technologies).

Cardiac differentiation of human iPSCs. Human iPSCs were cultured and differentiated to cardiomyocytes as described previously (*6, 7*). In brief, iPSC were plated on matrigel (BD Biosciences)-coated 6-well plates. Small molecule-based cardiac differentiation of human iPSCs was performed as described (*6*). From day 7 of cardiac differentiation, spontaneously contracting iPSC-CMs were

observed. Human iPSC-CMs were cultured in RPMI medium (Life Technologies) complemented with B27 supplement (Life Technologies). On cardiac differentiation day 20-25, iPSC-CMs were dissociated with TryplE (Life Technologies) and used for the respective experimental analysis. For Rab5-GFP overexpression, iPSC-CMs were transfected with CellLight™ Early Endosome-GFP (Thermo Fisher Scientific) following the manufacturer's instructions. When indicated, MUT iPSC-CMs were treated with 3μg/mL Rho activator II (RhoA II) (Cytoskeleton) overnight.

Immunohistochemistry. Human iPSC-CMs grown on matrigel-coated glass coverslips were subjected to immunohistochemistry using a previously described protocol (*7, 8*). Briefly, coverslips were fixed with 4% PFA in PBS for 20 min, permeabilized using Triton X-100 in PBS, and unspecific antigen sites were blocked with 5% BSA (Sigma-Aldrich) in PBS. iPSC-CMs were incubated with primary antibody against an early endosome marker protein EEA1 (Cell Signaling Technology), TnT (Abcam), α-actinin (Sigma), myosin light chain 2a (Synaptic Systems) at 4°C. Afterward, secondary antibodies Alexa Fluor 488 goat anti-rabbit antibody (Thermo Fisher Scientific), Alexa Fluor 568 donkey anti-mouse antibody (Thermo Fisher Scientific), or goat anti-rabbit antibody (Jackson Immuno Research, West Grove, PA, USA) custom-labelled with Abberior STAR RED (Abberior, Germany) were applied. Coverslips were mounted on glass slides using Fluromount G (SouthernBiotech) or Mowiol 4-88 with 0.1% 1,4-diazabicyclo[2.2.2]octane (DABCO) (Sigma Aldrich, St. Louise, MO, USA). Confocal images were taken with a plan apochromat (63x/1.40 oil DIC M27) objective using an inverted confocal microscope (LSM 710 Meta, Zeiss) using ZEN software (Zeiss).

SUPPLEMENTARY FIGURE LEGENDS

Figure S1. Characterization of iPSC-CMs.

A, Representative confocal images of TPM1-L185F (MUT1) iPSC-CMs and WT control (WT1) following immunostaining with sarcomeric α-actinin and troponin T antibodies. **B**, Representative confocal images of TnT-R141W (MUT2) iPSC-CMs and WT control (WT2) following immunostaining with sarcomeric α-actinin and troponin T antibodies. **C**, Representative confocal images of TPM1-L185F (MUT1) iPSC-CMs and WT control (WT1) following immunostaining with myosin light chain 2a and troponin T antibodies. **D**, Representative confocal images of TnT-R141W (MUT2) iPSC-CMs and WT control (WT2) following immunostaining with myosin light chain 2a and troponin T antibodies. Scale bar, 20 µm. ACTN2, sarcomeric α-actinin; TNNT2, cardiac troponin T; MYL7, myosin regulatory light chain 2.

Figure S2. Averages over z-layers (n=25-60) for image-based data after threshold application.

A-B, Overview on results for entire data population in WT1 versus MUT1 (DCM TPM1-L185F) iPSC-CMs. **C-D**, Overview on results for entire data population in WT2 versus MUT2 (DCM TnT-R141W) iPSC-CMs.

Figure S3. Robustness of distance distributions against sampling density.

A, Different choices of threshold (n_σ) are shown as weighted histograms. **B**, Different choices of sampling densities are shown as weighted histograms.

Figure S4. Dependence of distance distributions on cell size.

A, Averages over z-layers following application of threshold as in **Fig. 1D** for differently sized MUT1 iPSC-CMs. **B**, Weighted histograms for unscaled distance distribution corresponding to images shown in **A**. **C**, Weighted histograms for scaled distance distribution corresponding to images shown in **A**.

Figure S5. Distance distributions for all WT and MUT data sets.

A-B, Weighted histograms for scaled distance distributions in MUT 1 (TPM1-L185F) iPSC-CMs and the corresponding isogenic control, WT1. **C-D**, Weighted histograms for scaled distance distributions in MUT2 (TnT-R141W) iPSC-CMs and the corresponding isogenic control, WT2.

Figure S6. All scaled distance distributions for WT and MUT data sets without spherical modelling.

A-B, Scaled distance distributions for full data populations without spherical modelling indicate a bi-modal distribution in MUT iPSC-CMs compared to WT controls. **A**, MUT1 (TPM1-L185F) iPSC-CMs versus WT control (WT1); **B**, MUT2 (TnT-R141W) iPSC-CMs versus WT control (WT2) **C-D**, Averages of scaled distance distributions without spherical modelling for MUT iPSC-CMs compared to WT controls. **C**, MUT1 (TPM1-L185F) iPSC-CMs versus WT control (WT1); **D**, MUT2 (TnT-R141W) iPSC-CMs versus WT control (WT2)

Figure S7. Distance distributions for STED imaging data from WT and MUT iPSC-CMs.

A-B, Distance distributions for STED data populations compared to confocal imaging using the 1-sigma threshold. (**A**), WT2 and (**B**), MUT2 iPSC-CMs. Red, STED and blue, confocal distance distributions. n=1 experiment per cell line.

Figure S8. Intensity distributions for all STED data populations of MUT iPSC-CMs vs. WT.

A-B Intensity distributions for STED versus confocal data populations detected. (**A**), WT2 and (**B**), MUT2 iPSC-CMs. Red outline, full range of confocal data distribution detected; grey outline, full range of STED data distribution detected. n=1 experiment per cell line.

Figure S9. Application of threshold and scaled distance distributions for MUT iPSC-CMs following RhoA II treatment.

A-B Threshold application for confocal images of MUT2 (TnT-R141W) iPSC-CMs treated with control vehicle (**A**) or RhoA II (**B**); overview on results for entire data population. n=2 independent experiments and independent batches of cardiac differentiation. **C-D**, Weighted histograms for scaled distance distributions for full data populations without spherical modelling indicate a recovery of the bi-modal distribution in MUT2 (TnT-R141W) iPSC-CMs treated with RhoA II (**B**) vs. control vehicle (**A**); overview on results for the entire data population.

Figure S10. Scaled distance distributions for MUT iPSC-CMs following RhoA II treatment.

A, Scaled distance distributions for full data populations without spherical modelling indicate a recovery of the bi-modal distribution in TnT-R141W (MUT2) iPSC-CMs treated with RhoA II, compared to control vehicle. **B**, Averages of scaled distance distributions without spherical modelling for MUT2 iPSC-CMs treated with RhoA II, compared to control vehicle, using data shown in **A**. n=2 independent experiments using independent batches of cardiac differentiation, results corresponding to data shown in **Figure S9**. Control vehicle, n=42 cells; RhoA II, n=41 cells.

SUPPLEMENTAL MOVIES

Video S1: 3D movie of WT vesicular distributions calculated based on confocal imaging of early endosome immunostaining in WT1 iPSC-CMs.

Video S2: 3D movie of MUT vesicular distributions calculated based on confocal imaging of early endosome immunostaining in MUT1 iPSC-CMs.

REFERENCES

1. Y. Dai, N. Ignatyeva, H. Xu, R. Wali, K. Toischer, S. Brandenburg, C. Lenz, J. Pronto, F. E. Fakuade, S. Sossalla, E. M. Zeisberg, A. Janshoff, I. Kutschka, N. Voigt, H. Urlaub, T. B. Rasmussen, J. Mogensen, S. E. Lehnart, G. Hasenfuss, A. Ebert, An Alternative Mechanism of Subcellular Iron Uptake Deficiency in Cardiomyocytes. *Circ Res* **133**, e19-e46 (2023).
2. N. Sun, M. Yazawa, J. Liu, L. Han, V. Sanchez-Freire, O. J. Abilez, E. G. Navarrete, S. Hu, L. Wang, A. Lee, A. Pavlovic, S. Lin, R. Chen, R. J. Hajjar, M. P. Snyder, R. E. Dolmetsch, M. J. Butte, E. A. Ashley, M. T. Longaker, R. C. Robbins, J. C. Wu, Patient-specific induced pluripotent stem cells as a model for familial dilated cardiomyopathy. *Sci Transl Med* **4**, 130ra147 (2012).
3. H. Xu, R. Wali, C. Cheruiyot, J. Bodenschatz, G. Hasenfuss, A. Janshoff, M. Habeck, A. Ebert, Non-negative blind deconvolution for signal processing in a CRISPR-edited iPSC-cardiomyocyte model of dilated cardiomyopathy. *FEBS Lett*, (2021).
4. G. Chen, D. R. Gulbranson, Z. Hou, J. M. Bolin, V. Ruotti, M. D. Probasco, K. Smuga-Otto, S. E. Howden, N. R. Diol, N. E. Propson, R. Wagner, G. O. Lee, J. Antosiewicz-Bourget, J. M. Teng, J. A. Thomson, Chemically defined conditions for human iPSC derivation and culture. *Nat Methods* **8**, 424-429 (2011).
5. A. D. Ebert, K. Kodo, P. Liang, H. Wu, B. C. Huber, J. Riegler, J. Churko, J. Lee, P. de Almeida, F. Lan, S. Diecke, P. W. Burridge, J. D. Gold, D. Mochly-Rosen, J. C. Wu, Characterization of the molecular mechanisms underlying increased ischemic damage in the aldehyde dehydrogenase 2 genetic polymorphism using a human induced pluripotent stem cell model system. *Sci Transl Med* **6**, 255ra130 (2014).
6. X. Lian, C. Hsiao, G. Wilson, K. Zhu, L. B. Hazeltine, S. M. Azarin, K. K. Raval, J. Zhang, T. J. Kamp, S. P. Palecek, Robust cardiomyocyte differentiation from human pluripotent stem cells via temporal modulation of canonical Wnt signaling. *Proc Natl Acad Sci U S A* **109**, E1848-1857 (2012).
7. X. Lian, J. Zhang, S. M. Azarin, K. Zhu, L. B. Hazeltine, X. Bao, C. Hsiao, T. J. Kamp, S. P. Palecek, Directed cardiomyocyte differentiation from human pluripotent stem cells by modulating Wnt/beta-catenin signaling under fully defined conditions. *Nat Protoc* **8**, 162-175 (2013).
8. F. Lan, A. S. Lee, P. Liang, V. Sanchez-Freire, P. K. Nguyen, L. Wang, L. Han, M. Yen, Y. Wang, N. Sun, O. J. Abilez, S. Hu, A. D. Ebert, E. G. Navarrete, C. S. Simmons, M. Wheeler, B. Pruitt, R. Lewis, Y. Yamaguchi, E. A. Ashley, D. M. Bers, R. C. Robbins, M. T. Longaker, J. C. Wu, Abnormal calcium handling properties underlie familial hypertrophic cardiomyopathy pathology in patient-specific induced pluripotent stem cells. *Cell Stem Cell* **12**, 101-113 (2013).

Supplemental Figure 1

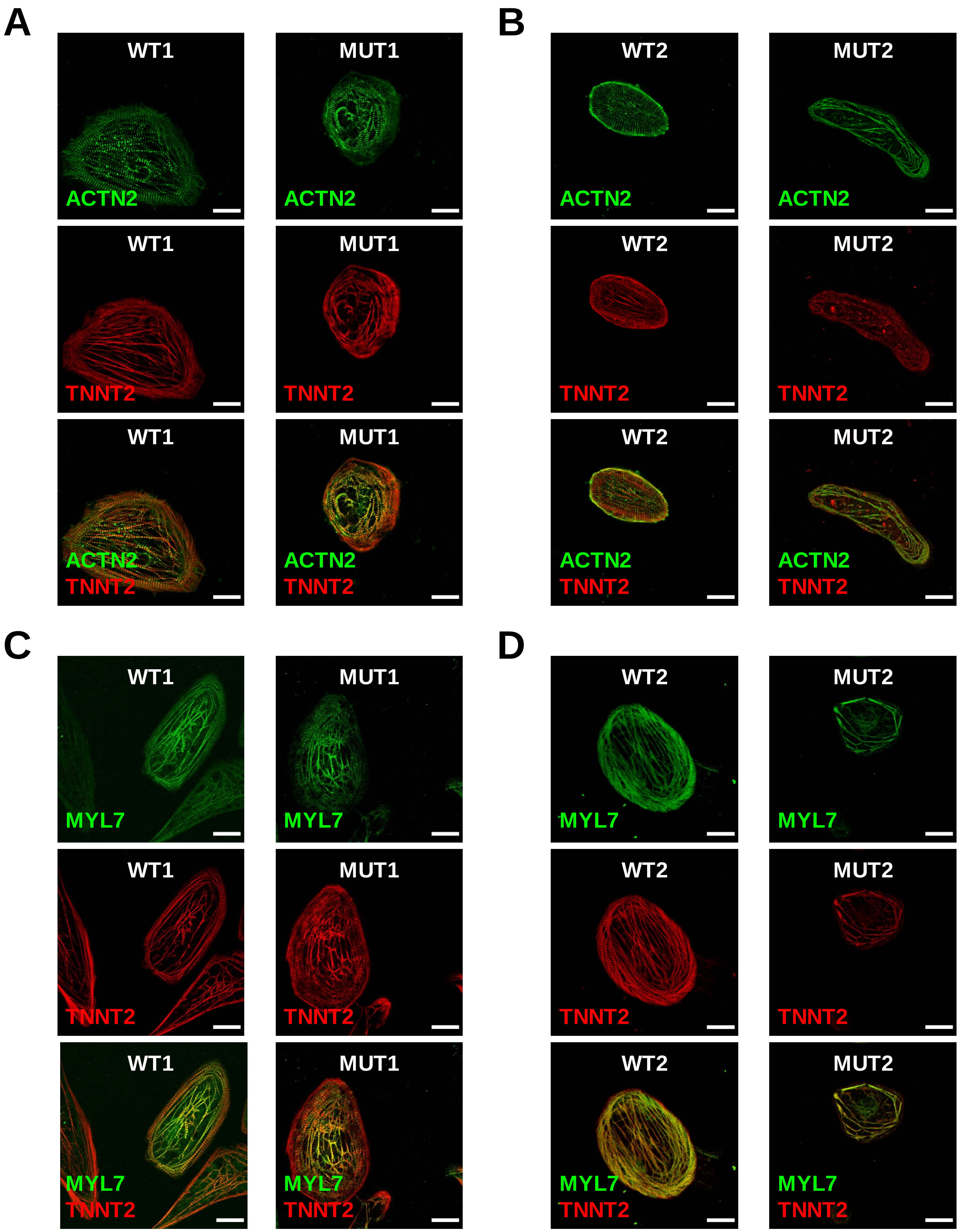

Supplemental Figure 2

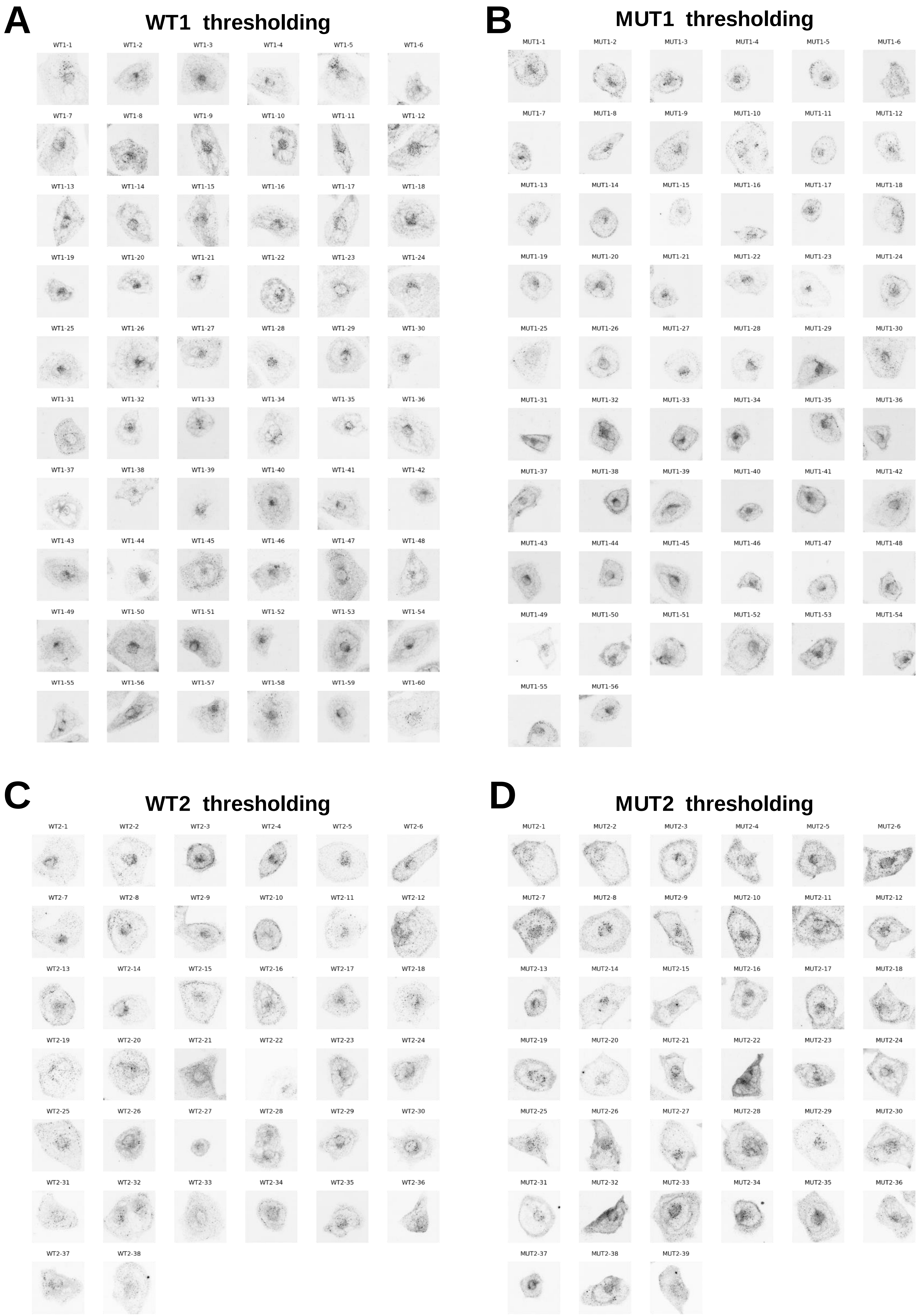

Supplemental Figure 3

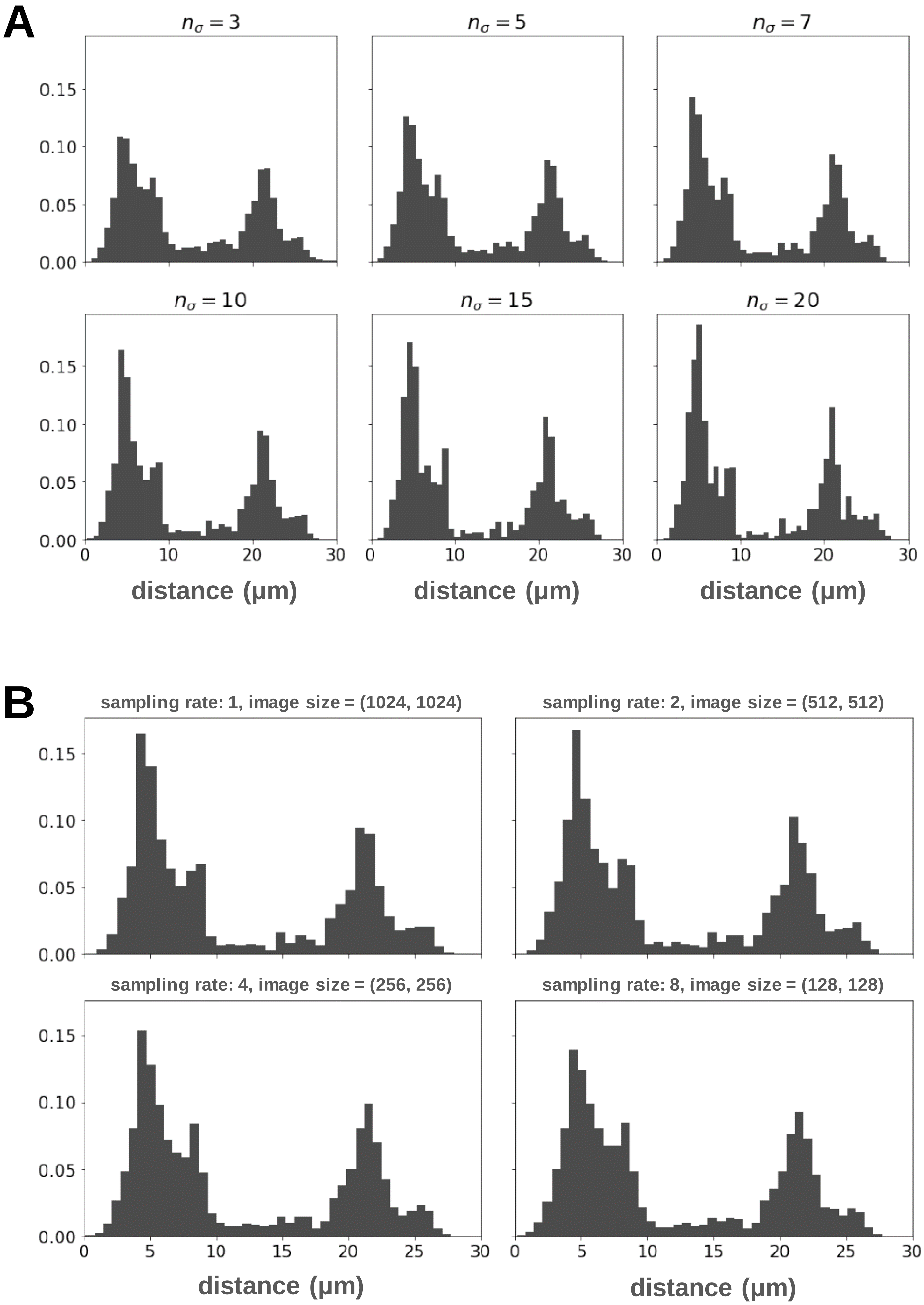

Supplemental Figure 4

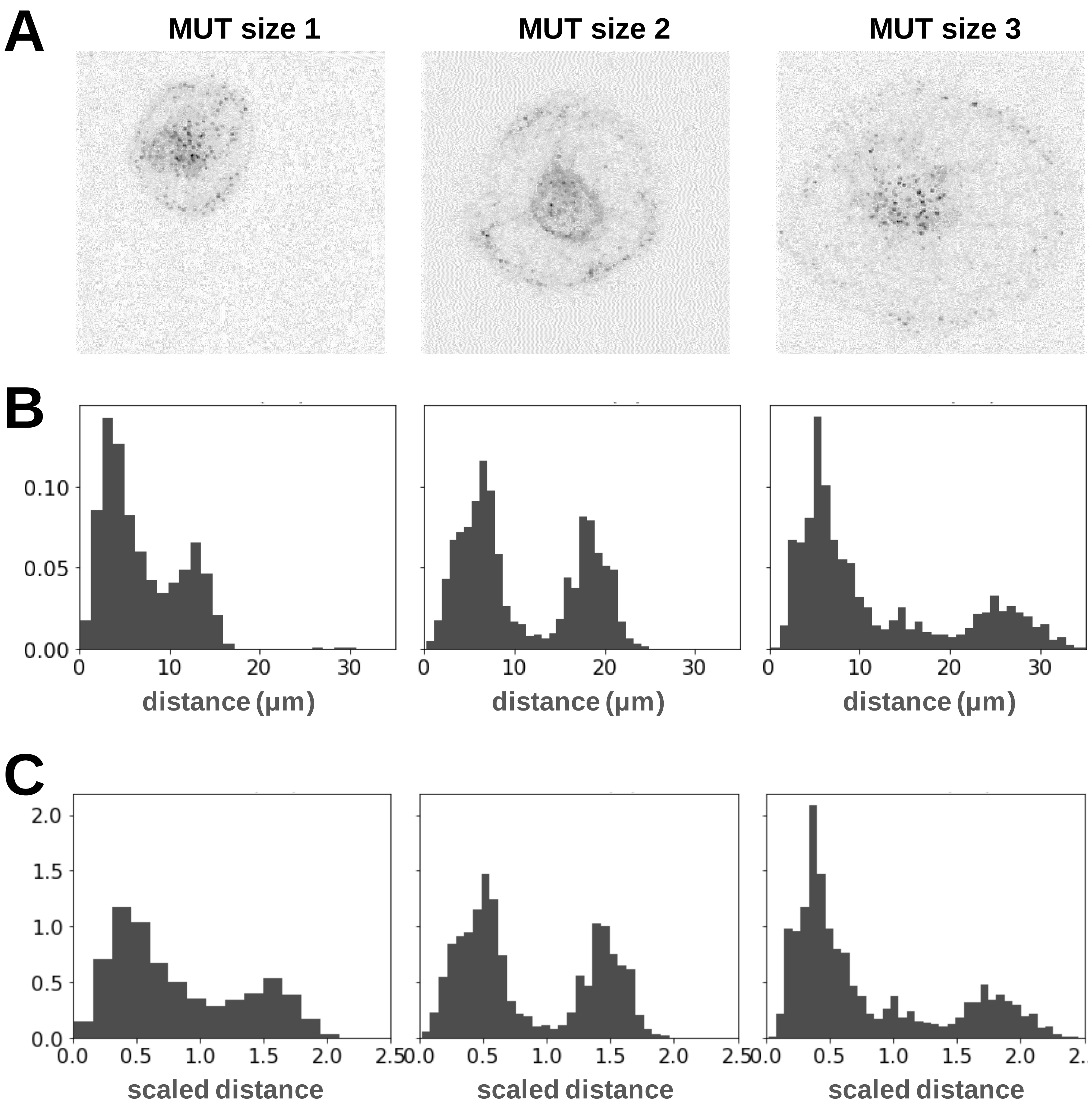

Supplemental Figure 5

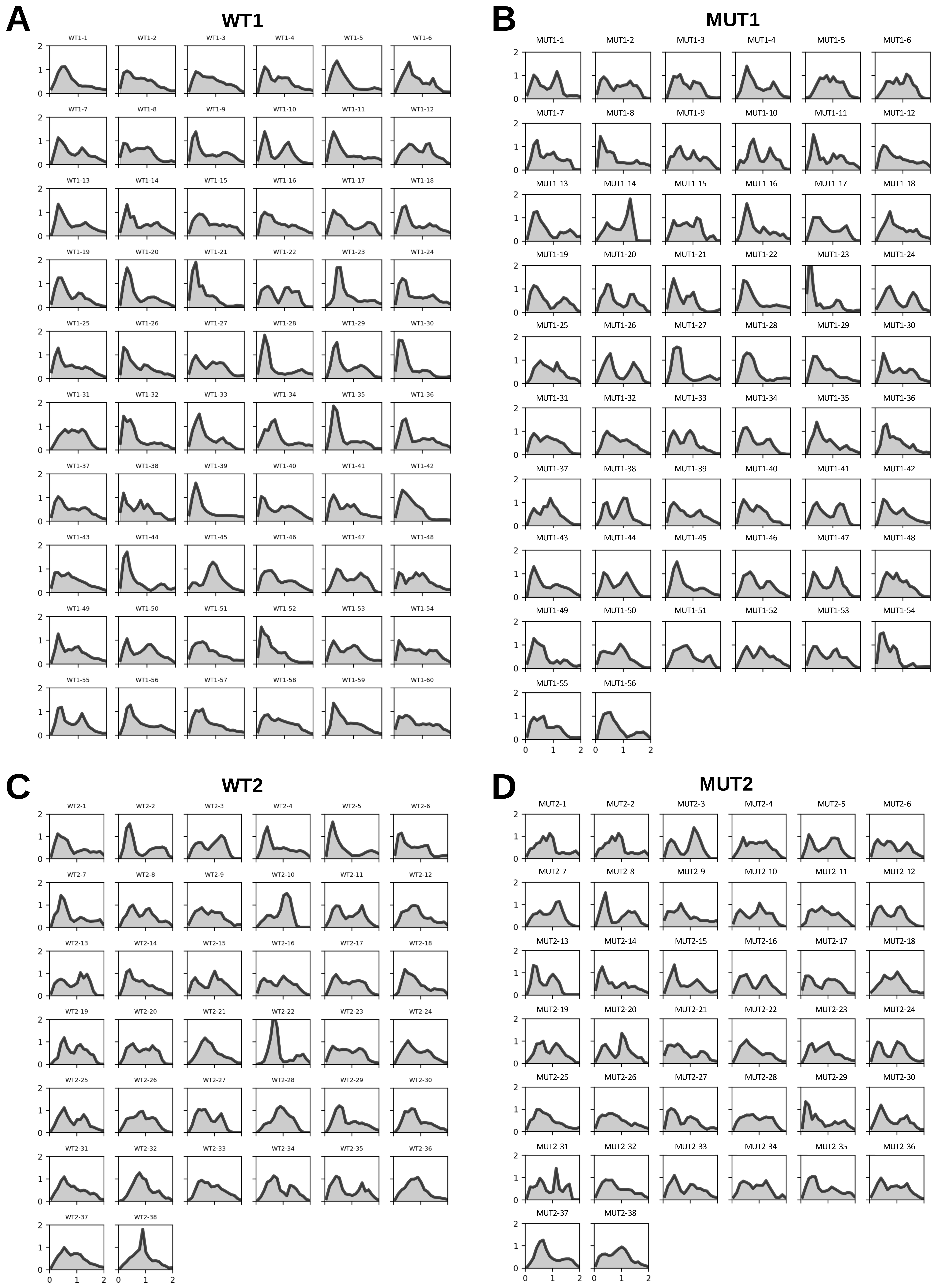

Supplemental Figure 6

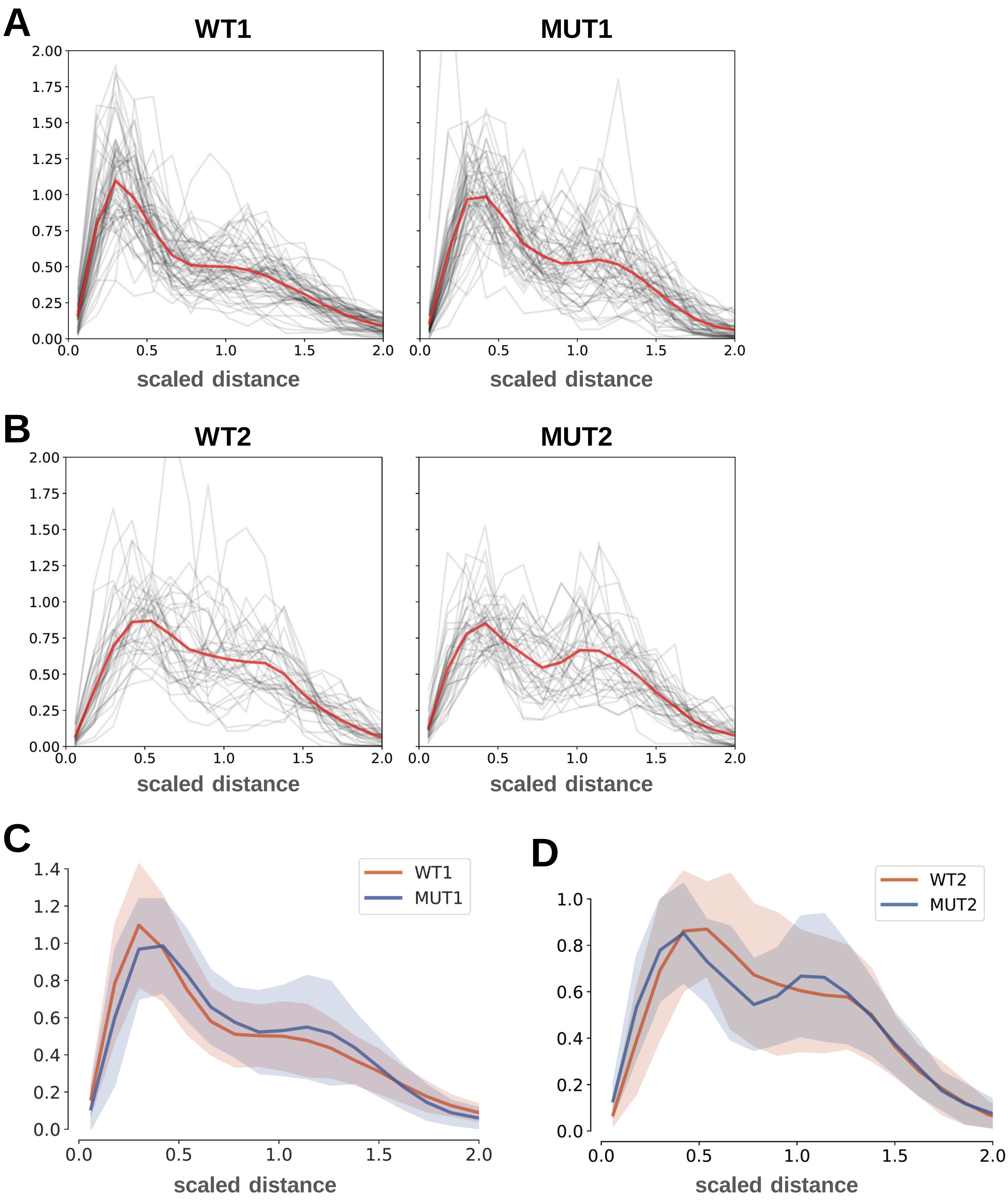

Supplemental Figure 7

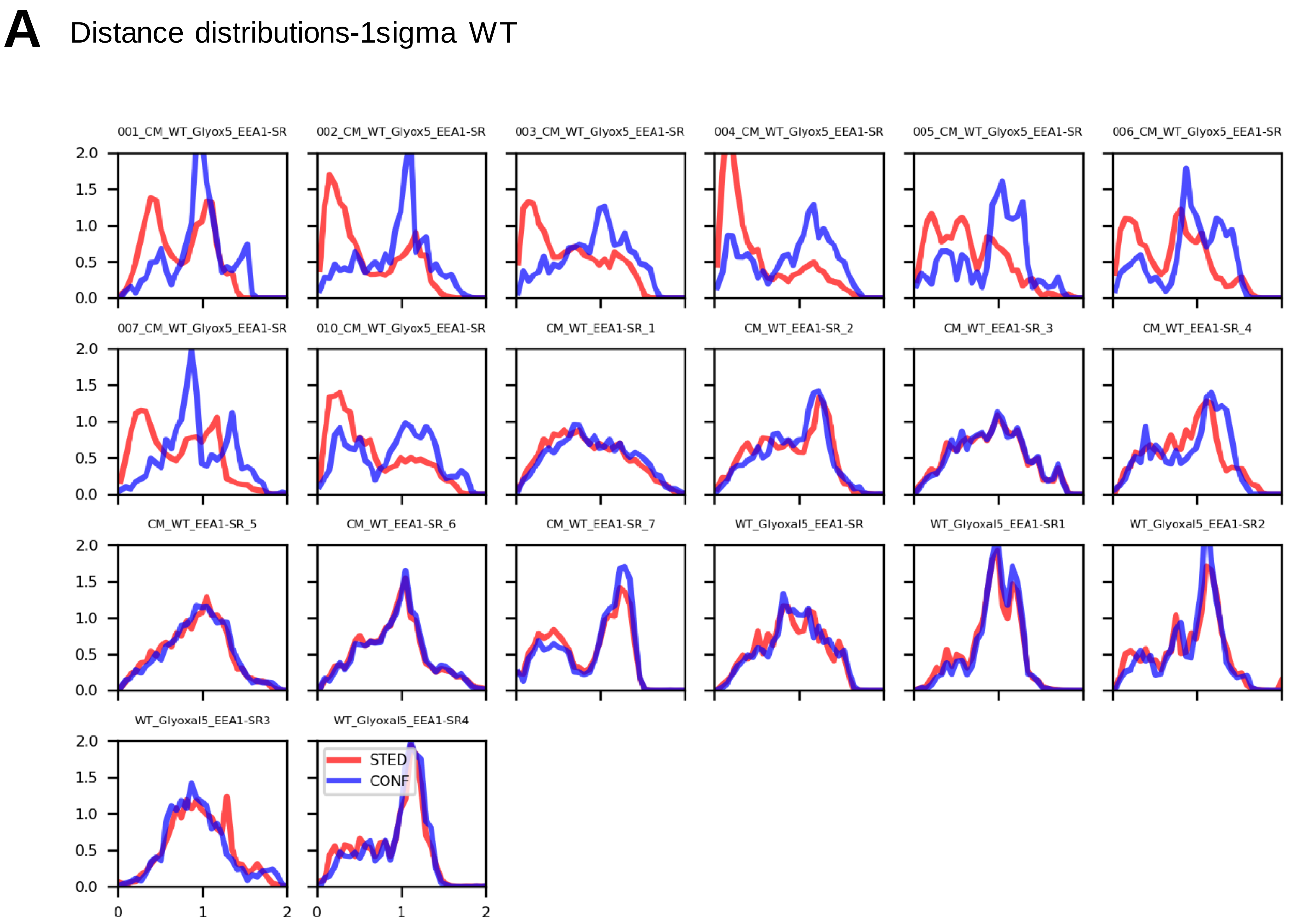

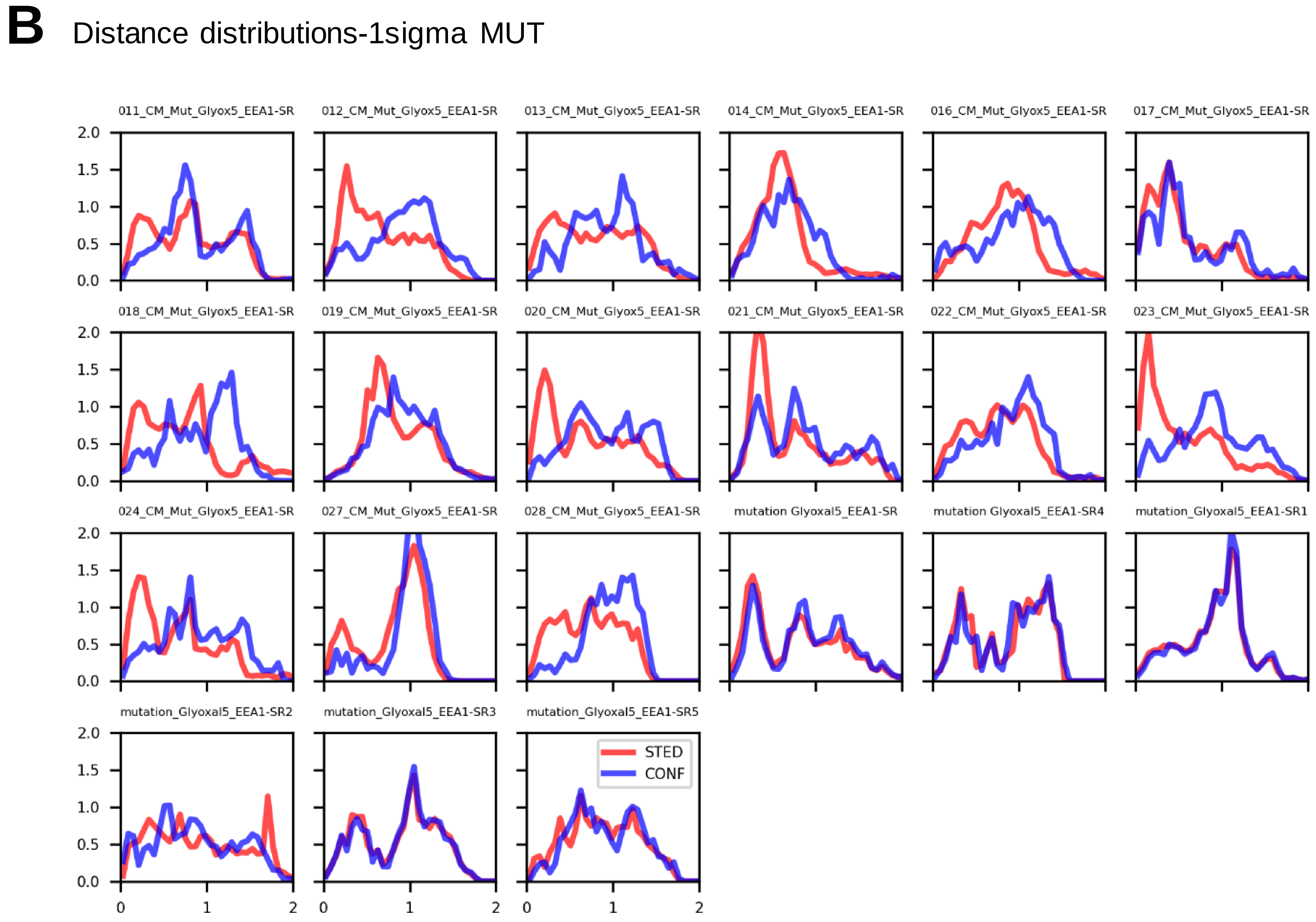

Supplemental Figure 8

A Intensity distributions WT

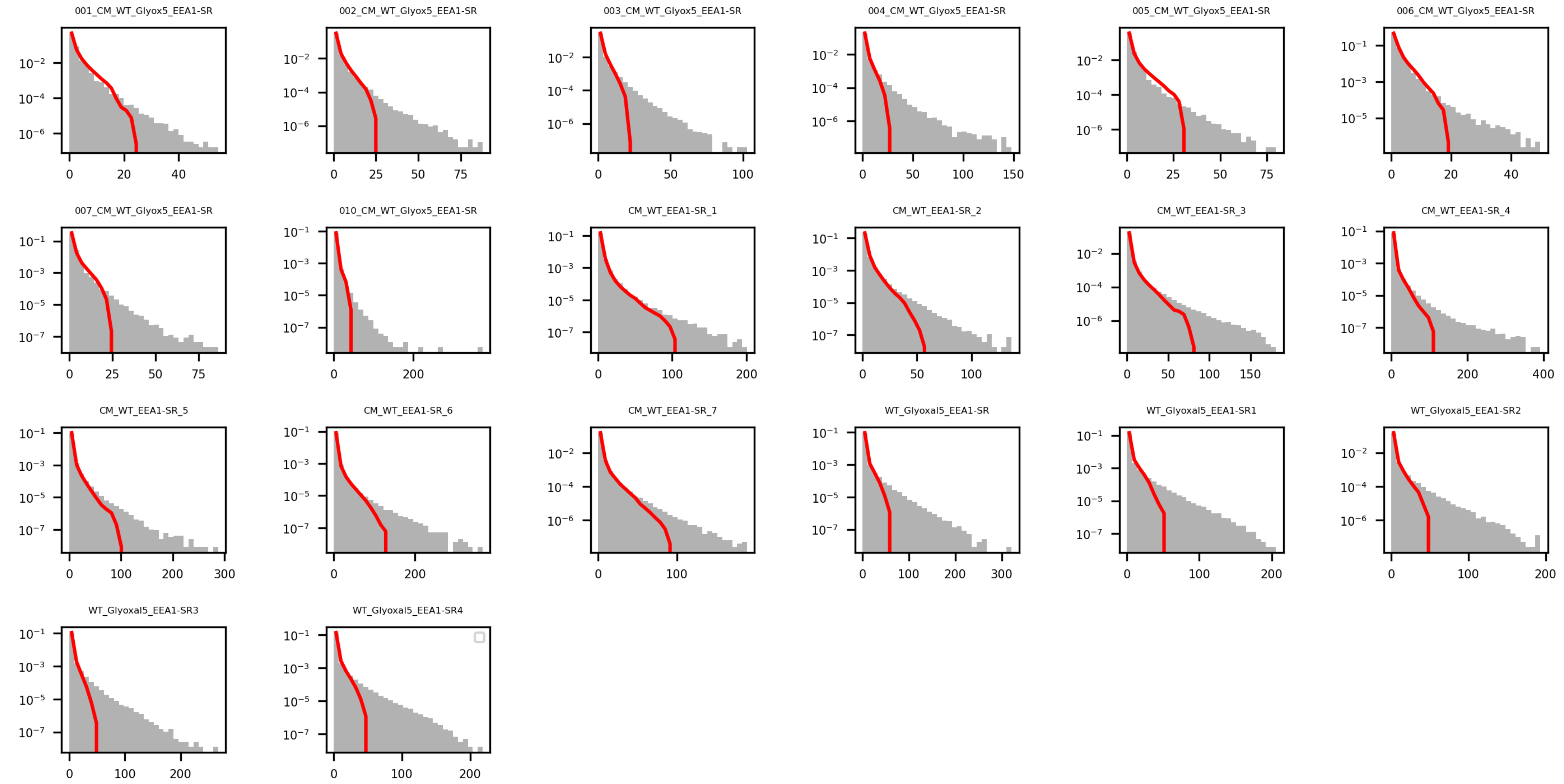

B Intensity distributions MUT

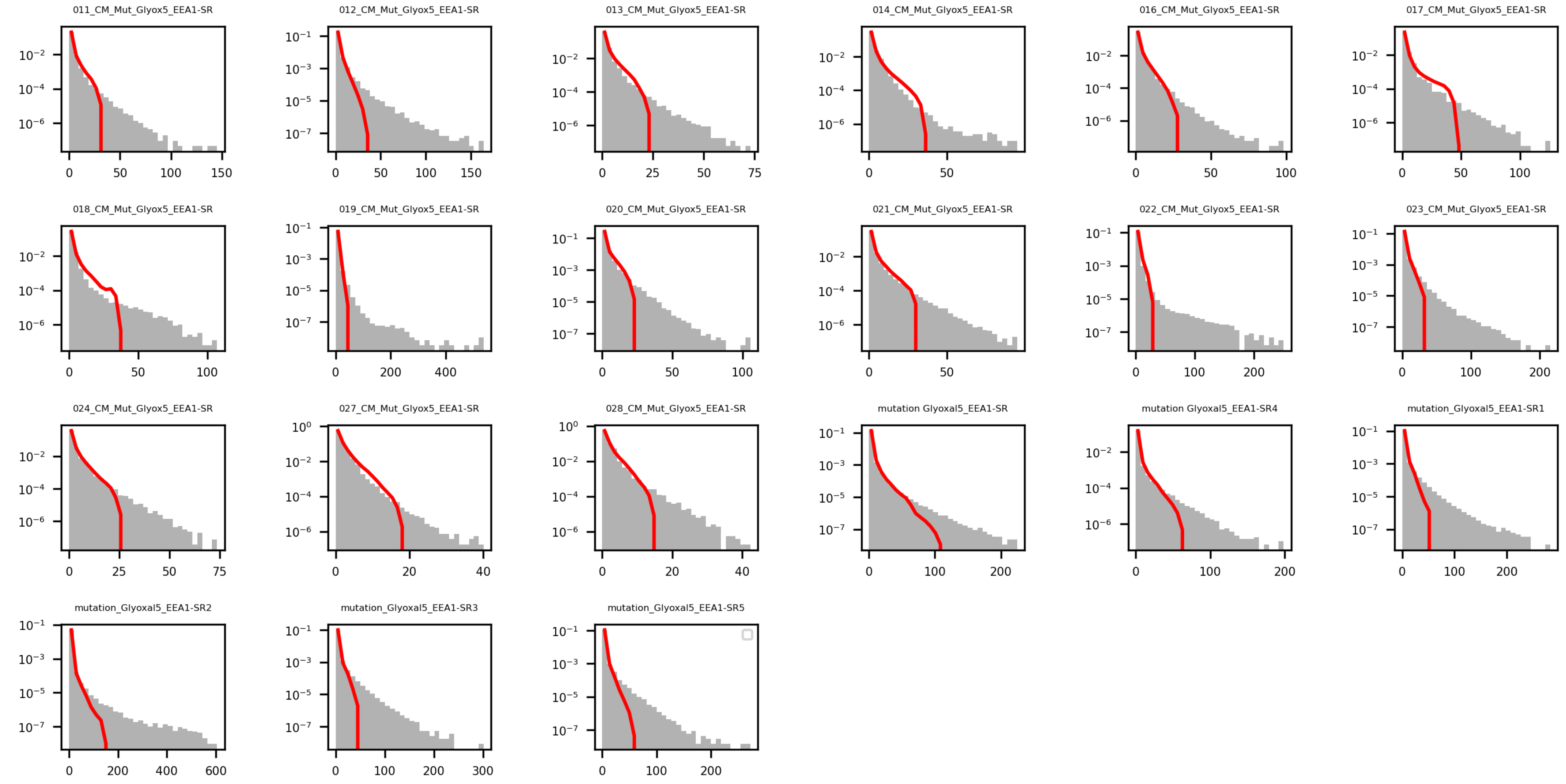

Supplemental Figure 9

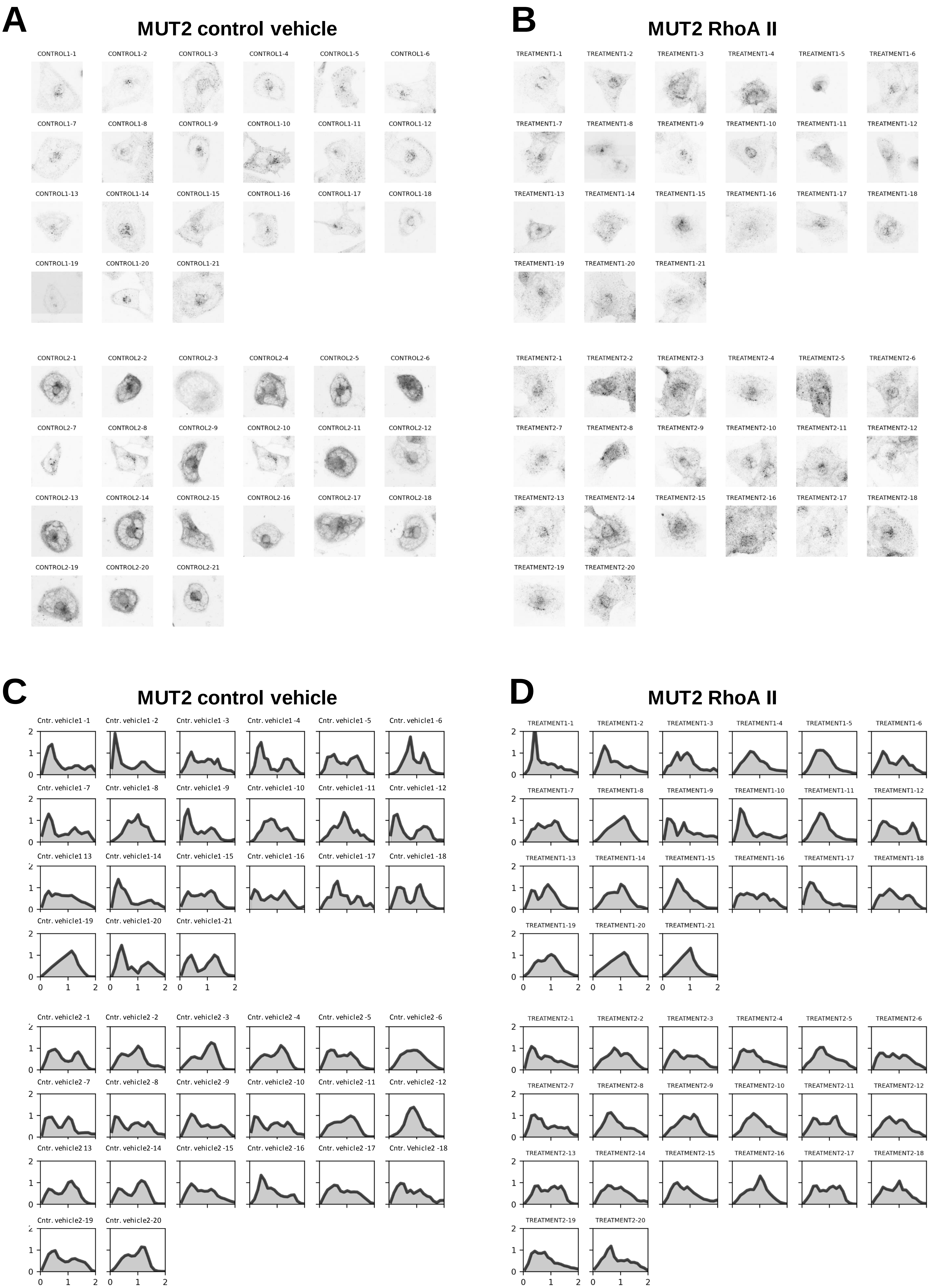

Supplemental Figure 10

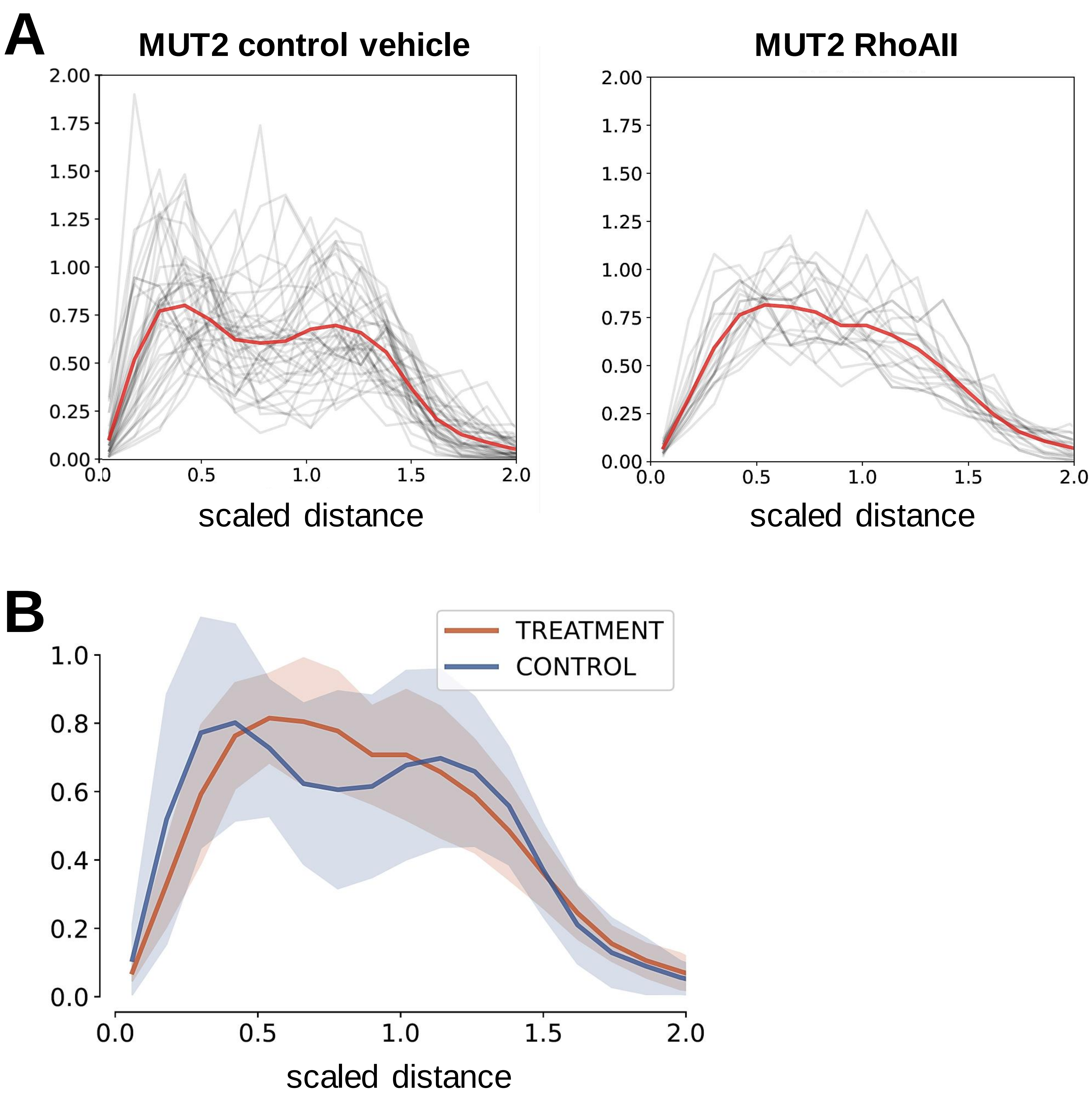

Supplement: Supplementary file 1 [file cells-13-00923-s001.zip › Saleem et al_Supplementary Materials.pdf]
